# Genome-wide analyses reveal a role of Polycomb in promoting hypomethylation of DNA methylation valleys

**DOI:** 10.1186/s13059-018-1390-8

**Published:** 2018-02-08

**Authors:** Yuanyuan Li, Hui Zheng, Qiujun Wang, Chen Zhou, Lei Wei, Xuehui Liu, Wenhao Zhang, Yu Zhang, Zhenhai Du, Xiaowo Wang, Wei Xie

**Affiliations:** 1 Center for Stem Cell Biology and Regenerative Medicine, MOE Key Laboratory of Bioinformatics, THU-PKU Center for Life Sciences, School of Life Sciences, Tsinghua University, Beijing, 100084 China; 2 Bioinformatics Division, TNLIST/MOE Key Laboratory of Bioinformatics, Center for Synthetic and System Biology, Department of Automation, Tsinghua University, Beijing, 100084 China

**Keywords:** DMV, DNA methylation valley, Polycomb, TET, Epigenetics, Methylome

## Abstract

**Background:**

Previous studies showed that the majority of developmental genes are devoid of DNA methylation at promoters even when they are repressed. Such hypomethylated regions at developmental genes are unusually large and extend well beyond proximal promoters, forming DNA methylation valleys (DMVs) or DNA methylation canyons. However, it remains elusive how most developmental genes can evade DNA methylation regardless of their transcriptional states.

**Results:**

We show that DMVs are hypomethylated in development and are highly conserved across vertebrates. Importantly, DMVs are hotspots of regulatory regions for key developmental genes and show low levels of deamination mutation rates. By analyzing a panel of DNA methylomes from mouse tissues, we identify a subset of DMVs that are dynamically methylated. These DMVs are strongly enriched for Polycomb-deposited H3K27me3 when the associated genes are silenced, and surprisingly show elevated DNA methylation upon gene activation. 4C-seq analyses indicates that Polycomb-bound DMVs form insulated and self-interacting chromatin domains. Further investigations show that DNA hypomethylation is better correlated with the binding of Polycomb than with H3K27me3. In support of a role of Polycomb in DMV hypomethylation, we observe aberrant methylation in DMVs in mouse embryonic stem cells deficient in the EED protein. Finally, we show that Polycomb regulates hypomethylation of DMVs likely through ten-eleven translocation (TET) proteins.

**Conclusions:**

We show that Polycomb promotes the hypomethylation of DMVs near key developmental genes. These data reveal a delicate interplay between histone modifiers and DNA methylation, which contributes to their division at distinct gene targets, allowing lineage-specifying genes to largely maintain DNA methylation-free at regulatory elements.

**Electronic supplementary material:**

The online version of this article (10.1186/s13059-018-1390-8) contains supplementary material, which is available to authorized users.

## Background

Animal development is strictly controlled by cell type- and stage-specific gene expression programs that are primarily regulated by transcription factors [1, 2]. On the other hand, epigenetic regulators play critical roles in maintenance of cellular memory once the cell identity is established [3]. DNA methylation is a major epigenetic mark regulating gene repression, genomic imprinting, and X chromosome inactivation [4, 5]. DNA methyltransferase 1 (DNMT1) recognizes hemi-methylated CpGs and methylates CpGs on the newly synthesized DNA during genome replication. This ensures the heritability of the methylation patterns in each cell lineage [6]. Two other methyltransferases, DNMT3a and DNMT3b, can conduct de novo DNA methylation. DNA methylation is essential for animal development, as mice deficient in *Dnmt1* or *Dnmt3* are embryonically lethal [7–9]. In addition to DNA methylation, chromatin modifications also play crucial roles in gene regulation and development [10]. For example, histone H3K4me3 is a mark that is associated with gene activity [11]. On the other hand, H3K27me3 is a repressive histone mark that is strongly linked to gene silencing [12]. Disruption of the H3K4me3 methyltransferases, such as mixed-lineage leukemia 1 (MLL1) and MLL2, or the Polycomb repressive complex 2 (PRC2), which is responsible for H3K27 methylation, frequently leads to embryonic lethality [10, 13].

Given the profound impact on animal development by ablation of DNMTs, DNA methylation has long been considered as a major repression mechanism of tissue-specific genes [6]. However, other studies as well as ours revealed that, upon cell differentiation, DNA methylation appears to be regulated only at a small number of promoters, whereas it is much more dynamic at enhancers [14–16]. DNA methylation is constantly absent at many promoters of key regulators for embryonic development regardless of their transcription activities. These promoters frequently show unusually large hypomethylated regions extending well beyond their proximal promoters, a pattern that we termed DNA methylation valley (DMV) [14]. Similar patterns were also identified by other groups as “broad non-methylated islands (NMIs)” [17] or “DNA methylation canyons” [18]. Interestingly, DMVs often include both CpG islands (CGIs), which are known to be constantly hypomethylated [19], and non-CGI regions. How DMVs are regulated and maintained as hypomethylated, and why they occur predominantly at key transcription factor genes remain elusive.

Notably, a large portion of DMVs are marked by H3K27me3 [14, 17, 18], which is consistent with the notion that developmental gene promoters are preferential targets of Polycomb [20]. It is believed that the repression mediated by H3K27me3 is easier to reverse, thus retaining developmental plasticity. DNA methylation, on the other hand, is considered as a more stable repression mechanism [6]. The fact that DMVs are strongly marked by H3K27me3 raises an interesting possibility that these two mechanisms may antagonize each other. Indeed, it has been shown that PRC2 binds poorly to nucleosomes with methylated DNA [21, 22]. As a possible consequence, depletion of DNA methylation leads to “spreading” of H3K27me3 into non-target regions and a decrease of H3K27me3 at Polycomb targets [23, 24]. Such spreading of H3K27me3 can repress retrotransposon activities in the absence of DNA methylation [25]. A single molecular analysis also revealed that H3K27me3 and DNA methylation seldom co-exist [26]. However, other studies also suggest that DNA methylation and H3K27me3 can co-occur in a genomic region-dependent manner, particularly in regions with low CG density [23, 27]. On the other hand, the effects of Polycomb and H3K27me3 on DNA methylation are less clear. Previously, it was shown that the depletion of H3K27me3 only alters the DNA methylation at a limited number of promoters [28]. However, this change of DNA methylation was determined using methylated DNA immunoprecipitation (MeDIP), a relatively low-resolution approach [29]. In this study, we sought to determine the molecular mechanisms underlying the regulation of hypomethylation in DMVs. Our results showed that DMVs are strongly enriched for transcription factor binding sites and are highly conserved in sequences. Furthermore, we found that Polycomb is required for the maintenance of DMV hypomethylation and regulates DNA methylation, likely through ten-eleven translocation proteins (TETs). We propose that Polycomb may promote hypomethylation of DMVs at key transcription factor genes and contribute to the fidelity of regulatory elements.

## Results

### DMVs are hypomethylated throughout development and are conserved across vertebrates

**Fig. 1:**
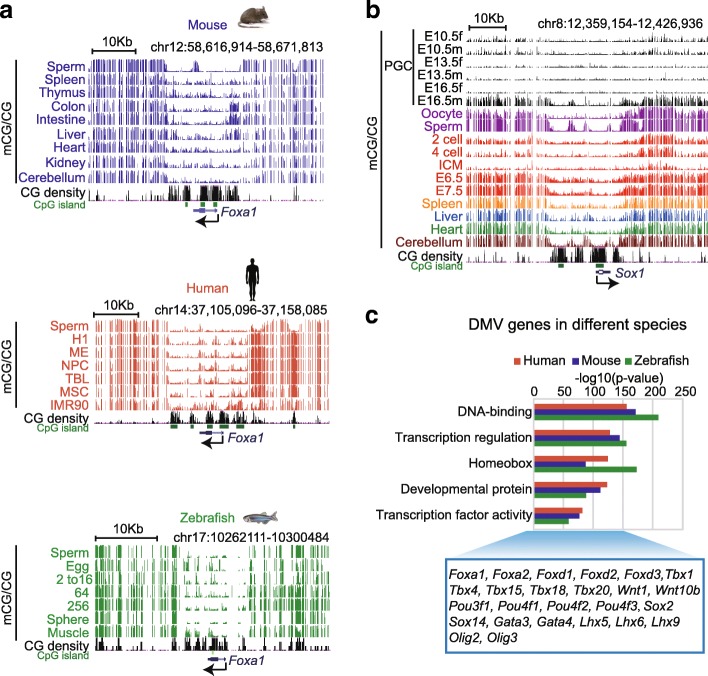
A global survey of DNA methylation valleys (DMVs) in various vertebrates. **a** University of California, Santa Cruz (UCSC) Genome Browser snapshots of various methylomes for a DMV near *Foxa1* in three vertebrates: mouse [15], human [14], and zebrafish [33]. *H1* human embryonic stem cell (hESC), *ME* mesendoderm, *NPC* neural progenitor cell, *TBL* trophoblast-like cell, *MSC* mesenchymal stem cell, *IMR90*, human fetal lung fibroblast cell line. **b** UCSC Genome Browser snapshot of a DMV near *Sox1* at different stages of mouse development. **c** Gene ontology analysis of DMV genes in different vertebrates (human, mouse, and zebrafish). Examples of transcription factors shared among all three vertebrates are listed below

Previously, DMVs or DNA methylation canyons were identified in a number of human and mouse cell types [14, 18]. We aimed to determine if DMVs are also broadly present in different species and across developmental stages. By examining a panel of 16 mouse tissue methylomes [15], we found widespread DMVs in these tissues using a method described previously [14] (Methods) (Fig. 1a, Additional file 1: Figure S1A), with numbers ranging from 696 to 1454 (Additional file 1: Figure S1B, Additional file 2: Table S1). To examine if DMVs are present throughout the mouse developmental cycle, we examined DNA methylome data in primordial germ cells (PGCs) (E10.5, E13.5, and E16.5) [30], spermatozoa, oocytes, and early embryos [31]. We found that DMVs (pooled from all mouse tissues) are largely hypomethylated across developmental stages (Fig. 1b, Additional file 1: Figure S1C). It is worth noting that we recently also determined DNA methylomes in mouse postimplantation lineages and found that a portion of DMVs are relatively hypermethylated in E5.5 epiblast and extraembryonic tissues, although their overall levels of DNA methylation are still low compared to the genome average [32]. Finally, we wondered if DMVs are conserved in other vertebrate species. By investigating the methylomes from zebrafish [33], we also identified DMVs with associated genes strongly enriched for developmental genes and genes coding transcription factors similar to those of human and mouse (Fig. 1a, c, Additional file 3: Table S2). Taken together, these data suggest that DMVs are hypomethylated throughout developmental periods and are highly conserved in vertebrates.

### DMVs are hotspots of TF binding sites

**Fig. 2:**
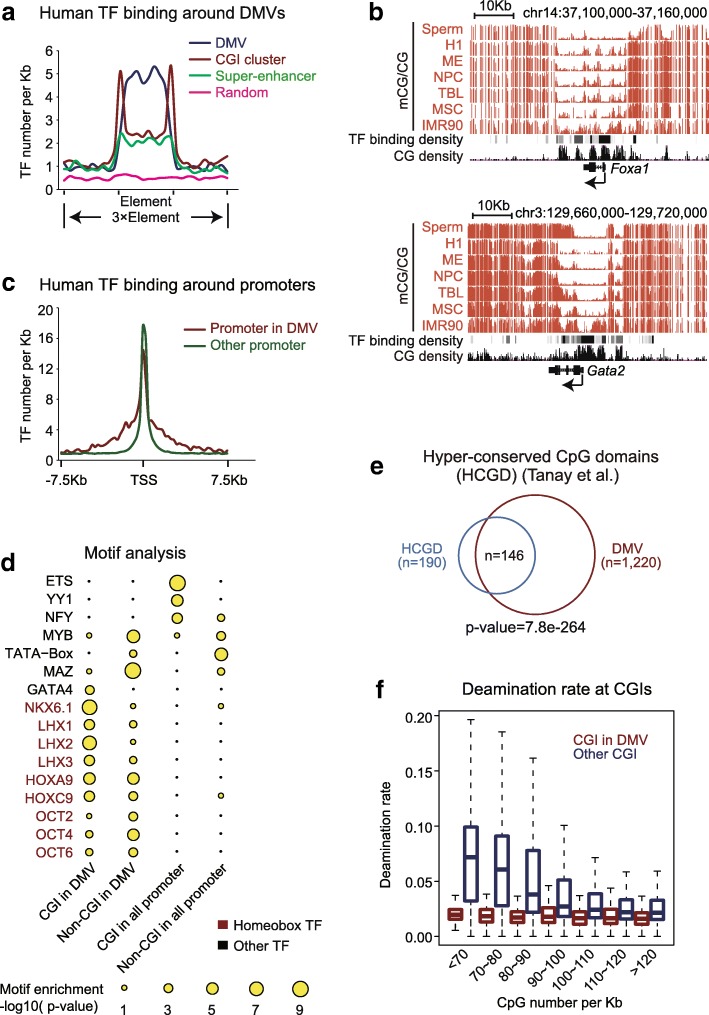
DMVs are hotspots of transcription factor (*TF*) binding sites. **a** The average distribution of TF binding sites (per kb) around human DMVs or CpG island (*CGI*) clusters based on ChIP-seq data from ENCODE [35]. Random sequences with the same lengths as DMVs in the genome were used as a control. CGI clusters were defined as described previously [14]. Super-enhancers were defined in human embryonic stem cells as previously described [37]. **b** UCSC Genome Browser snapshots of DNA methylomes for sperm [85] and various cell types [14] and TF binding density [35] in human DMVs near *Foxa1* and *Gata2*. **c** The average distribution of TF bindings around transcription start sites (*TSSs*) in human [35] for promoters in DMVs or other promoters. **d** TF motif analysis of different regions in the human genome CGI in DMVs, non-CGI in DMVs, CGI in all promoters, and non-CGI in all promoters). The scale of the *circles* represents motif enrichment. *Red* represents homeobox TFs. **e** Venn diagram showing the overlap between DMVs and hyper-conserved CpG island domains (HCGDs) [41] in human. **f** Boxplot showing deamination rates [42] for human CGIs inside or outside DMVs classified based on their CG densities

An intriguing question that remains is why developmental genes require large hypomethylated domains at promoters. We reasoned that these key regulator genes are in turn strictly controlled by other transcription regulators. Using human methylomes we reported previously [14] and transcription factor chromatin immunoprecipitation sequencing (ChIP-seq) datasets from the Encyclopedia of DNA Elements (ENCODE) [34–36], we found that transcription factor binding sites are densely present in DMVs and that the binding frequencies decrease sharply outside of DMVs (Fig. 2a, b). Interestingly, DMVs overall contain even more transcription factor binding sites compared to super-enhancers [37] and simple CGI clusters [14]. This is contributed by both CGIs and, to a lesser extent, non-CGI regions of DMVs (Additional file 1: Figure S2A). The ranges of transcription factor binding sites are much broader for the promoters of DMV genes than for non-DMV genes (Fig. 2c). Such results were also similarly observed when using a dataset of mouse transcription factor binding sites determined by ChIP-seq [38] (Additional file 1: Figure S2B). Consistent with the notion that transcription factor bindings are associated with nucleosome depletion [39], we found that DMV regions are occupied by fewer nucleosomes than their surrounding regions (Additional file 1: Figure S2C). Interestingly, by searching for transcription factor motifs present in DMVs, we found that CGI and non-CGI regions in DMVs are enriched for motifs for many homeobox transcription factors such as NKX, LHX, HOX, and OCT factors and other developmental regulators such as GATA factors (Fig. 2d). As a control, this was not observed for all promoters in the genome (Fig. 2d). Hence, these data indicate that DMVs are hotspots of regulatory elements for key developmental genes.

The high density of TF binding in DMVs indicates that developmental genes may require complex regulation that involves a large set of regulatory factors. It is tempting to speculate that the constant hypomethylation of DMVs may be crucial for maintaining the plasticity of gene expression by avoiding DNA methylation at regulatory regions. On the other hand, methylcytosines are known as hotspots of mutations, as DNA methylation can lead to deamination of Cs and subsequent conversion to Ts [19]. Recent efforts of large-scale cancer genome sequencing also confirmed that the most prominent mutations in cancer are C-to-T mutations in the CpG context [40]. Therefore, the presence of DMVs may help decrease the risks of spontaneous deamination mutations. Indeed, a previous study identified 190 hyper-conserved CpG island domains (HCGDs) in the human genomes [41]. Strikingly, we found that 146 out of 190 (77%) HCGDs are present in DMVs [14] (Fig. 2e). In fact, DMVs are more conserved than CGI clusters [14] and super-enhancers [37] (Additional file 1: Figure S2D). By examining a published dataset of sequence deamination rate in the human genome derived from multiple species sequence comparison [42], we indeed observed low rates of deamination in DMV regions compared to their surrounding regions (Additional file 1: Figure S2E). This is not simply due to CG density, as both CGIs and non-CGIs in DMVs show lower deamination rates than loci outside of DMVs with similar CG density levels (Fig. 2f, Additional file 1: Figure S2F). Therefore, these data raise a possibility that the hypomethylation of DMVs may help ensure expression plasticity and sequence fidelity at these regulatory elements.

### Analyses of DNA methylation and gene expression revealed dynamic regulation of DMVs

**Fig. 3:**
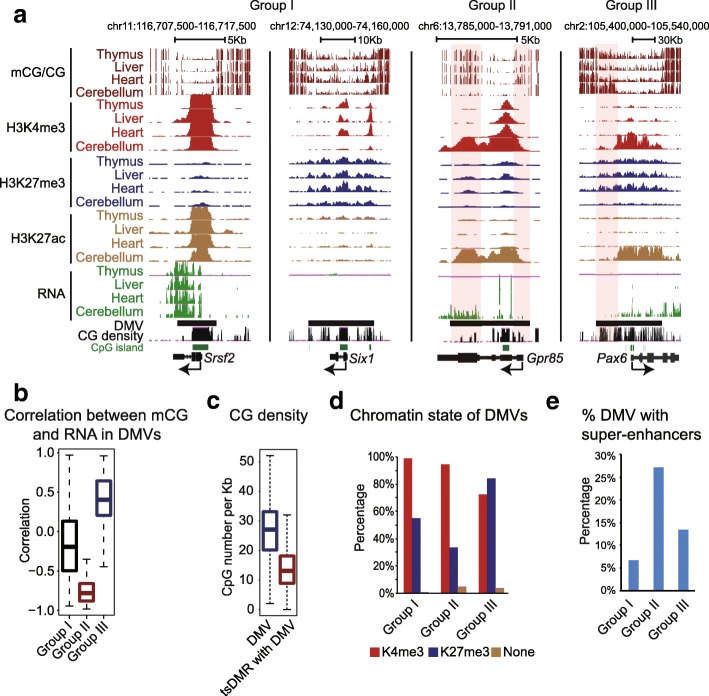
Identification of three groups of DMVs. **a** UCSC Genome Browser snapshots of DNA methylomes, histone modifications, and RNA levels near group I DMVs (*Srsf2*, *Six1*), a group II DMV (*Gpr85*), and a group III DMV (*Pax6*) in mouse tissues. Regions with dynamic DNA methylation are shaded. **b** Boxplot showing the correlation coefficients between DNA methylation and gene expression for three groups of DMVs in mouse tissues. **c** Boxplot showing the CG density for DMVs or tissue-specific differentially methylated regions (tsDMR) within DMVs. **d** The chromatin state (presence of H3K4me3 and/or H3K27me3) is shown for different groups of DMVs. **e** Percentages of DMVs with super-enhancers are shown as bar graphs for different groups of DMVs

Intriguingly, while the majority of human DMVs remain hypomethylated in various cell types, we also found that a number of DMVs clearly show variable DNA methylation across mouse tissues (Fig. 3a, right two panels). Interestingly, using hierarchical clustering, we showed that the DNA methylation levels in mouse DMVs could correctly group tissues with similar origins (Additional file 1: Figure S3A), suggesting that DNA methylation variations in DMVs are linked to lineage identities. We further classified all mouse tissue DMVs into three groups (Fig. 3a, b) (Methods). Group I (*n* = 1580) shows constant DNA methylation levels in DMVs across all tissues regardless of the expression levels of their associated genes. Group II (*n* = 132) shows dynamic DNA methylation levels across different tissues, demonstrating lower DNA methylation when associated genes are activated. This is consistent with the notion that transcription is generally associated with DNA hypomethylation near promoters [6]. The third group of DMVs (*n* = 34) also shows dynamic DNA methylation levels across different tissues. However, to our surprise, these DMVs demonstrated higher DNA methylation when the associated genes are expressed. Notably, this increased DNA methylation is largely limited to non-CGIs in DMVs and loci outside of promoters (Fig. 3a). To characterize these hypermethylated regions, we took advantage of a study that identified tissue-specific differentially methylated regions (tsDMRs) across these mouse tissues [15]. Indeed, we found that tsDMRs in these DMVs preferentially reside in low-CG regions (Fig. 3c). By examining a panel of histone modification ChIP-seq data in mouse tissues [43], we found that groups I and III DMVs are preferentially enriched for high CpGs (Additional file 1: Figure S3B) and H3K27me3 (Fig. 3d), both of which are known to occupy developmental regulator promoters [12, 13]. Indeed, these two groups, but not group II, are enriched for transcription factor genes (Additional file 1: Figure S3C). Notably, group III contains much larger DMVs compared to the other two groups (Additional file 1: Figure S3B), which may contribute to its dynamic nature. As a comparison, group I DMVs are smaller, and their constant hypomethylation may result from strict protection mechanisms near proximal promoters including those through CpG islands [19]. By contrast, group II DMV regions show tissue-specific H3K4me3 peaks and clustered histone H3K27ac peaks (Fig. 3a), a feature that resembles that of super-enhancers [44]. Indeed, by identifying super-enhancers in various tissues using a previously described method [37], we found that group II DMVs are significantly enriched for super-enhancers (27%) compared to groups I and III (~5% and 14%, respectively) (Fig. 3e). This is also consistent with the previous observation that enhancers are generally hypomethylated [16]. In sum, these data revealed that a subset of DMVs are dynamically regulated. It is intriguing why DMVs marked by H3K27me3 are preferentially hypomethylated including those in groups I and III. Thus, we mainly focused on these DMVs in the subsequent analyses.

### Polycomb is required for maintenance of hypomethylation in DMVs

**Fig. 4:**
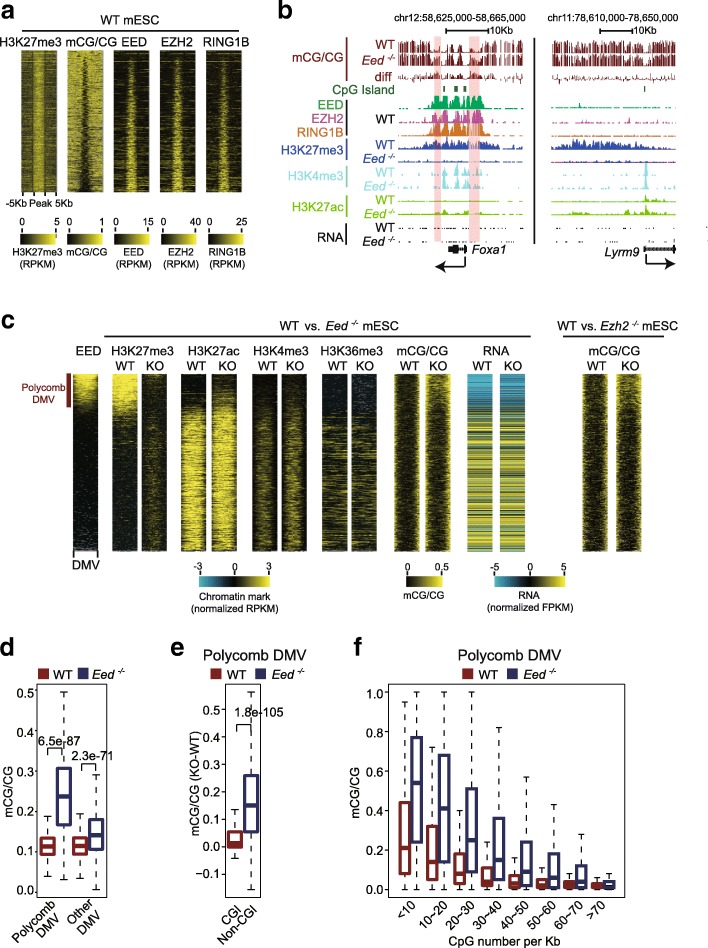
Polycomb is required for maintenance of hypomethylation in DMVs. **a** Heatmaps representing the corresponding level of DNA methylation and binding intensity of EED, EZH2, and RING1B [45] for large K27me3 peaks (> 5 kb, the minimal length of DMVs). Peaks were sorted by the DNA methylation levels in DMVs. An analysis of all H3K27me3 peaks yielded similar results (data not shown). **b** The epigenetic landscape is shown for Polycomb-bound DMV gene *Foxa1* and H3K27me3-marked non-DMV gene *Lyrm9*. Regions with elevated DNA methylation in *Eed* ^-/-^ mESCs are *shaded*. **c** Heatmaps representing the level of DNA methylation, H3K27me3, H3K27ac, H3K4me3, H3K36me3 within DMVs, and RNA level for DMV genes in WT and *Eed* ^-/-^ mESCs. DNA methylation level in *Ezh2* ^-/-^ mESCs is also shown. All DMVs are normalized to the same length. Levels of chromatin marks were Z-score-normalized. Fragments per kilobase per million mapped reads (*FPKMs*) of RNA level for WT and *Eed* ^-/-^ mESCs were quantile-normalized and log-transformed. EED-bound DMVs were defined as Polycomb DMVs. **d** Boxplots showing the DNA methylation level of Polycomb DMVs and other DMVs in WT and *Eed* ^-/-^ mESCs. *P* values (two-sided *t* test) are shown. **e** Boxplots showing the DNA methylation change (knockout *(KO)-WT*) for CpG islands (*CGI*) and non-CpG island (*non-CGI*) regions within Polycomb DMVs. *P* value (two-sided *t* test) is shown. **f** Boxplot showing DNA methylation level in Polycomb DMVs for regions with different CG densities in WT and *Eed* ^-/-^ mESC lines

We sought to determine what causes the hypomethylation of H3K27me3-marked DMVs. Notably, as increased DNA methylation in DMVs is accompanied by the loss of H3K27me3 upon gene activation for group III DMVs (Fig. 3a), we reasoned that the Polycomb protein complex may be important for keeping DNA methylation-free at these DMVs. Intriguingly, we found that the binding of the Polycomb proteins (EED, enhancer of zeste homolog 2 (EZH2) [45], RING1B [45]) is more correlated with hypomethylation in DMVs than H3K27me3, as regions marked only by H3K27me3 but not Polycomb do not show DNA hypomethylation (Fig. 4a, b, Additional file 1: Figure S4A). Therefore, these data indicate that the hypomethylation of DMVs may be related to Polycomb instead of H3K27me3. To test the role of Polycomb in DMV hypomethylation, we investigated DNA methylation using MethylC-sequencing (MethylC-seq) in wild-type (WT) mouse embryonic stem cells (mESCs) [14] and mESCs deficient in *Eed*, a component of the PRC2 complex [46]. The loss of *Eed* was validated by probing H3K27me3 using western blots (Additional file 1: Figure S4B) and ChIP-seq (Fig. 4c). Strikingly, we observed widespread elevation of DNA methylation in DMVs in *Eed* ^-/-^ cells (Fig. 4c). We also confirmed that no significant expression change of DNMTs was observed in these cells (data not shown). These observations were further verified using mESCs deficient in *Ezh2*, another key component of Polycomb (Fig. 4c), and several other WT mESC methylomes to avoid cell line methylation variations (Additional file 1: Figure S4C). Such DNA methylation increase is less evident for non-Polycomb-targeted DMVs (Fig. 4d). Importantly, our analyses showed that approximately 60% of group III DMVs identified in mouse tissues show hypermethylation in *Eed* ^-/-^ mESCs (compared to 18% and 10% of groups I and II, respectively), suggesting a similar function of PRC2 in maintaining hypomethylation in tissues and mESCs. Notably, regions with elevated methylation in the *Eed*-deficient cells are again limited to non-CGI regions in DMVs (Fig. 4e). In regions with lowest CG density, DNA methylation can increase more than 0.3 (Fig. 4f). Notably, CGI regions are maintained DNA methylation-free by several mechanisms including H3K4me3, a histone mark that is mutually exclusive with DNA methylation [19]. Therefore, these results suggest that Polycomb and other factors at CGIs collaboratively maintain the hypomethylated state of DMVs.

### DMVs are insulated self-interacting domains

It remains unclear how the loss of Polycomb leads to increased DNA methylation in H3K27me3-marked DMVs. DNMTs can be recruited to transcribed gene bodies through H3K36me3 [47–49], raising the question of whether the elevated DNA methylation may result from derepression of developmental genes in the absence of Polycomb. RNA-seq analyses revealed that developmental genes generally showed no or only weak reactivation upon *Eed* knockout in our data (Fig. 4b, Additional file 1: Figure S4D), as also shown in a previous study [50]. This is true for both genes with DMVs that show hypermethylation and those that remain unmethylated (Additional file 1: Figure S4E). In addition, we did not observe acquisition of H3K36me3 [51] in DMVs in *Eed* ^-/-^ cells (Fig. 4c, Additional file 1: Figure S4F), suggesting that the increased DNA methylation is likely not due to increased transcription or H3K36me3. On the other hand, the PRC1 complex can compact chromatin both in vivo and in vitro [52, 53]. Furthermore, EED also interacts with histone deacetylases [54], and loss of *Eed* leads to increased histone acetylation [55–57] (Fig. 4c). It is possible that the loss of *Eed* may lead to decompacted chromatin that allows the access of DNA methyltransferases to Polycomb-targeted DMVs. To determine if this is the case, we performed circular chromosome conformation capture sequencing (4C-seq) to investigate the chromatin architecture around DMV regions in mESCs and *Eed* ^-/-^ cells. Interestingly, for two DMVs we examined (*Pax6* and *Nkx2-2,* where large numbers of restriction enzyme cutting sites are available), the bait regions within the DMVs show strong interactions with other regions inside DMVs, but not regions outside of DMVs (Additional file 1: Figure S5A). Conversely, regions outside of DMVs demonstrate strong interactions with nearby regions but not those within DMVs. Similar observations were made for two additional DMVs, *Skor1* and *Ebf3* (data not shown). This is consistent with a recent study using 5C-seq published as this manuscript was in preparation [58]. Interestingly, the local insulated structure can still be observed around *Pax6* and *Nkx2-2* upon the loss of *Eed* in mESCs as determined through 4C-seq (Additional file 1: Figure S5A). As a control, expected interaction changes were observed at the HoxB region between WT and *Eed* knockout (KO) mESCs [59] (Additional file 1: Figure S5B). The interactions between DMVs and other Polycomb targets over distance are also lost in *Eed* KO mESCs (Additional file 1: Figure S5A) as reported previously [59–62]. Notably, Kundu et al. also showed that, upon the loss of *Phc1* (a component of the PRC1 complex) in mESCs, the compaction domains at Polycomb targets are strongly affected at the *Hox* loci, but to a much lesser extent at the *Pax6* locus [58]. The change of chromatin structure at *Pax6* became much more pronounced when cells underwent differentiation or when *Ring1b* (a key component of PRC1) was absent [58]. It is possible that the moderate structure changes for DMVs were not captured by 4C-seq due to the limited sensitivity. Therefore, these data indicate that Polycomb regulates DMV domains in a locus-specific and complex-specific manner. Taken together, these results showed that developmental genes and their putative regulatory elements form close interactions within DMVs prior to gene activation. Yet, this self-interacting structure is not entirely dependent on PRC2, at least in undifferentiated mESCs.

### Polycomb likely maintains hypomethylation of DMVs through TETs

**Fig. 5:**
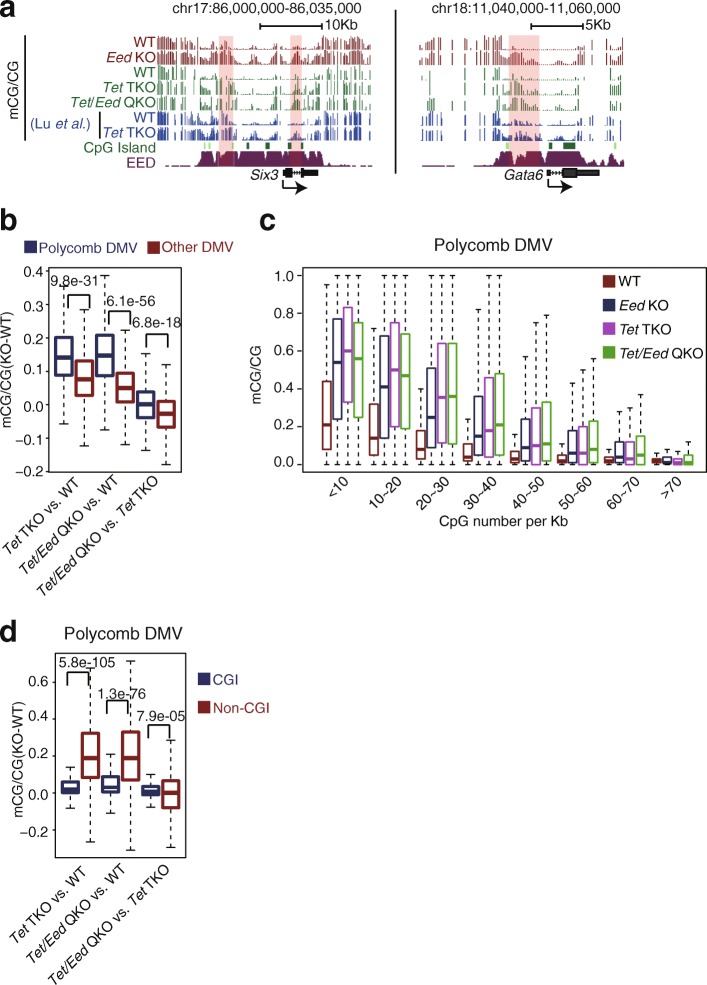
Polycomb likely regulates hypomethylation of DMVs through TETs. **a** UCSC Genome Browser snapshots of DNA methylation near DMV genes *Six3* and *Gata6* in WT, *Tet* TKO, and *Tet*/*Eed* QKO mESCs. Regions with elevated DNA methylation are *shaded. Tet* TKO: *Tet1/2/3* triple knockout, *Tet*/*Eed* QKO: *Tet1/2/3/Eed* quadruple knockout. **b** Boxplots showing the DNA methylation change (KO-WT) of Polycomb DMVs and other DMVs. *P* values (two-sided *t* test) are shown. **c** Boxplots showing DNA methylation level for regions within Polycomb DMVs with different CG densities in WT, *Eed KO*, *Tet* TKO, and *Tet/Eed* QKO mESC lines. **d** Boxplots showing the DNA methylation change (KO-WT) of CpG islands and non-CpG island regions within Polycomb DMVs. *P* values (two-sided *t* test) are shown

On the other hand, it was reported that TETs regulate demethylation of DMVs and promote cell differentiation [32, 63, 64]. Moreover, TET1 and PRC2 can form a complex [65]. TET1 can facilitate recruitment of Polycomb [66], and PRC2 knockdown also leads to downregulation of 5-hydroxymethylcytosine (5hmC) [65]. Therefore, we asked if Polycomb may regulate DMVs through TET proteins. By analyzing the methylome of *Tet1/2/3* triple KO *(Tet* TKO) mESCs [67], we found that Polycomb-bound DMVs preferentially show an increase of DNA methylation compared to other DMVs after the loss of TETs, which is very similar to the result when *Eed* is absent (Additional file 1: Figure S6A). The methylated regions are also limited to non-CGI regions in *Tet* TKO cells (Additional file 1: Figure S6B). To examine if Polycomb regulates DNA methylation in a similar or distinct pathway compared to TETs, we generated *Tet* TKO and further *Tet*/*Eed* quadruple KO (*Tet*/*Eed* QKO) mESC lines. Knockout of *Tets* and *Eed* was validated by genotyping (data not shown) and the absence of 5hmC and H3K27me3, respectively, in these cells (Additional file 1: Figure S6C, D). Interestingly, we observed a similar DNA methylation increase in *Tet* TKO and *Tet*/*Eed* QKO cells (Fig. 5a). Polycomb DMVs preferentially show an increase of DNA methylation in *Tet*/*Eed* QKO cells (Fig. 5b), with the increased DNA methylation primarily found in regions of low CG density (Fig. 5c) outside of CGIs (Fig. 5d). Importantly, DNA methylation in DMV did not show further increase after the loss of EED in *Tet* TKO cells, suggesting that Polycomb may regulate DMVs in the same pathway as TETs. Thus, these data indicate that Polycomb likely regulates DNA methylation in DMVs through TETs.

## Discussion

**Fig. 6:**
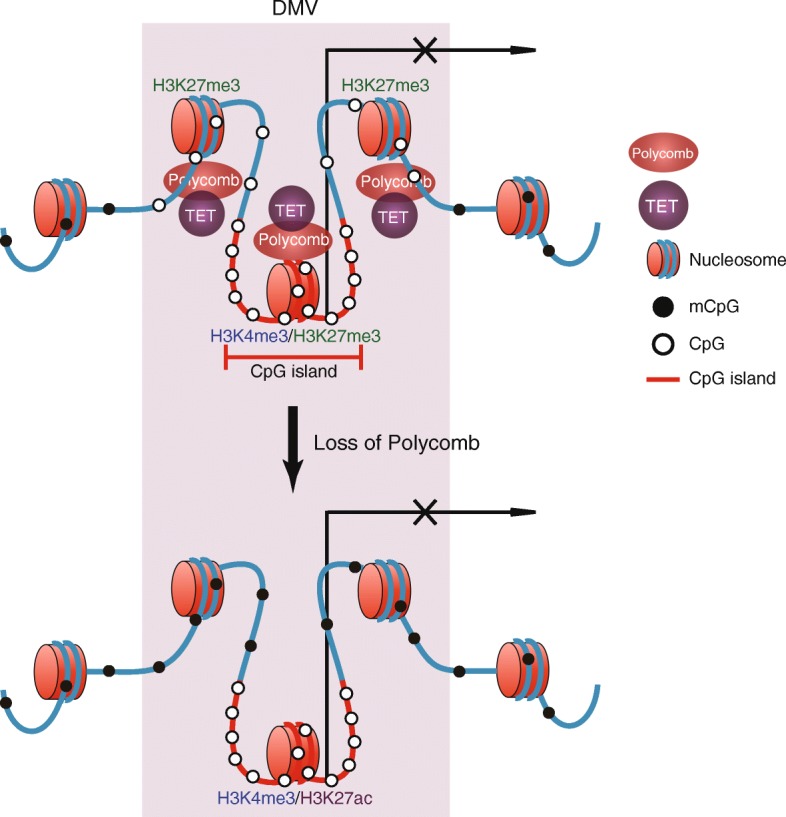
A model for the role of Polycomb in maintenance of DMV hypomethylation. Polycomb-targeted DMVs have lower nucleosome occupancy and higher CG density than their surrounding regions. They tend to form insulated structures from their surrounding methylated regions. In WT mESCs, Polycomb maintains DMV hypomethylation in non-CGI regions, likely through recruiting TETs. The absence of Polycomb leads to increase of DNA methylation in non-CGI regions and also increase of H3K27ac in CGI regions [56]. Genes near DMVs remain silenced during this process. CGIs in DMVs are maintained DNA methylation-free, presumably due to additional protection mechanisms including H3K4me3

The developmental programs are controlled by both transcription factors and epigenetic regulators. DNMTs and Polycomb are two key epigenetic regulators that maintain the silencing of lineage-specifiers [6]. Our previous work found that these two pathways primarily regulate distinct sets of genes [14]. Particularly, promoters of developmental genes are preferentially marked by H3K27me3, but they largely maintain hypomethylated DNA over long distances, forming DNA methylation valleys (DMVs). By contrast, DNA methylation is dynamically regulated at the promoters of a small subset of pluripotency genes and genes restricted in terminally differentiated tissues [14]. How DMVs are maintained methylation-free is not fully understood. In this study, we first showed that DMVs are hotspots of transcription factor binding and demonstrate high sequence fidelity during evolution. DMVs also display a distinct chromatin structure by forming insulated and self-interacting domains. A further systematic analysis of DMVs in mouse tissues showed that while the majority of DMVs remain constantly hypomethylated, a subset of them, especially those large DMVs, are dynamically regulated. The first class of dynamic DMVs are preferentially marked by super-enhancers, and their hypomethylation is likely associated with dense binding of transcription factors [44]. The second class, on the other hand, is preferentially marked by strong enrichment of H3K27me3. Surprisingly, the second class shows loss of H3K27me3 but elevated DNA methylation when nearby genes are expressed. Consistently, *Eed* knockout in mESCs also leads to elevated DNA methylation in DMVs, supporting a role of Polycomb in maintaining the hypomethylation of DMVs (Fig. 6). This likely reinforces the antagonism between Polycomb and DNA methylation, given that DNA methylation also restricts H3K27me3 to promoters [23, 24, 68]. Upon DNA methylation depletion, H3K27me3 would spread to other regions, leading to decrease of H3K27me3 at development gene promoters. Our data also showed that this is likely true for EED as well (Additional file 1: Figure S6E). This antagonism may ensure the proper division of epigenetic regulators in their targets in the genome.

How does Polycomb prevent DNA methylation in DMVs? Interestingly, we found that there exist regions in mESCs that are occupied by H3K27me3 but not Polycomb. These regions are still hypermethylated, suggesting that Polycomb, but not H3K27me3, is more likely to be involved in hypomethylation of DMVs. This is consistent with the previous studies showing that DNA methylation and H3K27me3 can indeed co-exist, particularly in regions with low CG densities [23, 27]. Polycomb can compact chromatin both in vitro and in vivo [52, 57, 58], raising an interesting question: Does Polycomb-mediated chromatin compaction possibly restrict the entrance of DNA methyltransferases? Indeed, DMV domains were shown to be more accessible in cells deficient in various Polycomb complex components in a perhaps locus-dependent manner [58]. This model, however, would presumably allow entrance of both DNMTs and TETs to DMVs. On the other hand, TET proteins were shown to be involved in demethylating DMVs [18, 32, 63, 65]. TET1 and PRC2 can form a complex, and their functions are interdependent [65, 66]. Our data further showed that loss of *Eed*, *Tet,* or both results in a similar increase of DNA methylation of DMVs, suggesting that Polycomb may regulate hypomethylation of DMVs through TET proteins (Fig. 6).

What are the functions of DMVs at developmental genes? Developmental genes tend to be regulated by a large number of transcription factors, which preferentially bind DMVs near these genes (Fig. 2a, b). DMVs often include both CpG island regions and non-CpG island regions. While CpG island regions are known to resist DNA methylation [19], non-CpG island regions in the genome are prone to DNA methylation. Exposing these TF binding sites to DNA methylation may possibly restrict the plasticity of gene expression in development. Alternatively, hypomethylation may reduce the risks of spontaneous deamination mutations caused by DNA methylation. Interestingly, a hallmark of cancer cells is aberrant promoter DNA methylation at developmental genes and Polycomb targets [69–72]. In line with this, many DMVs are preferentially marked by DNA methylation instead of H3K27me3 in cancer [14]. Curiously, the switch of repressive mechanisms occurs even though these genes are frequently silenced in both normal and cancer cells [73]. It is tempting to speculate that that this change of repression mechanism may be associated with increased mutation rates. Consistently, the C-to-T mutation is the most frequent mutation in cancer [40]. Both H3K4 and H3K27 methyltransferases are frequently dysregulated in cancer [74, 75]. Notably, developmental transcription factors including HOX genes are known to retain significantly higher portions of CGs in their coding regions compared to the genome average [76]. This, however, is not observed in organisms with no or a very low level of DNA methylation, such as drosophila. The unmethylated CpG islands may further help recruit Polycomb, which in turn promotes hypomethylation of DMVs, therefore creating a positive feedback loop. Finally, it is worth noting that Polycomb and TETs are only partially responsible for keeping DMV methylation-free. Future studies are needed to identify additional regulators that safeguard DMVs and, in particular, the high-CG regions that they contain. Taken together, our results revealed a possible role of Polycomb in the hypomethylation of DMVs at key developmental genes to maintain developmental plasticity and sequence fidelity of regulatory elements.

## Conclusions

One key question in epigenetics and development is: What is the exact role of DNA methylation in silencing lineage regulators? Despite the widespread presence of DNA methylation in the genome, the majority of developmental genes are in fact present in large constitutively hypomethylated regions, or DNA methylation valleys (DMVs). Here, we showed that DMVs are hotspots of transcription factor bindings and are highly conserved across vertebrates. Our 4C-seq data revealed that DMVs are insulated and self-interacting domains, indicating that developmental genes and their regulatory elements are restricted in local territory away from neighbor regions. Finally, we showed that Polycomb regulates DNA methylation in DMVs likely by recruiting the TET proteins. Our study not only highlights the important role of Polycomb in maintaining DNA methylation-free at regulatory elements of developmental genes, but it also unveils the mechanisms for the functional divisions of epigenetic regulators in controlling lineage specification.

## Methods

### Generation of knockout mESC lines

*Tet* TKO cells (R1) and *Tet*/*Eed* QKO (R1) were generated using CRISPR/Cas9. All single guide RNAs (sgRNAs) were designed using an online tool (http://crispr.mit.edu/) [77]. *Tet* genes were knocked out using one pair of sgRNAs and *Eed* using two pairs of sgRNAs. The sequences of sgRNAs were included in Table 1. The plasmid pX330-U6-Chimeric_BB-CBh-hSpCas9-PuroR (modified from pX330) [77] co-expressing Cas9 and sgRNA was used to transfect cells. Each pair of sgRNAs was phosphorylated, annealed, and ligated to a linearized vector. mESCs were transfected using plasmids with sgRNAs in Lipofectamine 3000 (L3000008, Thermo Fisher Scientific, Waltham, MA, USA) following the manufacturer’s instructions. Puromycin with a final concentration of 1 μg/mL was added to screen the transfected cells. After 24-h selection, about 50 cells were separated into a 96-well dish and cultured for about 1 week. Single clones were picked out and lysed with buffer K (36 μL ddH2O + 4 μL 10 × TBS + 0.4 μL 50% Tween 20 + 0.2 μL protease K) at 56 °C for 45 min and 95 °C for 15 min before genotyping.

The sgRNA targeted regions were as follows:

- *Tet1* chr10:62296286-62296377
- *Tet2* chr3:133148483-133151645
- *Tet3* chr6:83352674-83354788
- *Eed* chr7:97118816-97120844

### Cell culture

All mESCs were passaged every 48–72 h in Dulbecco’s modified Eagle’s medium (DMEM, Gibco) supplemented with 15% fetal bovine serum (FBS, HyClone), 1% non-essential amino acids (Gibco), 0.5% 2-mercaptoethanol (Gibco), 1% penicillin/streptomycin (Millipore, Bedford, MA, USA), 0.01% leukemia inhibiting factor (LIF) (Millipore), and 1% glutamine (Gibco) on gelatinized plates.

### MethylC-seq library generation and sequencing

We mixed 5 μg of genomic DNA isolated from mESCs with 25 ng unmethylated cl857Sam7 Lambda DNA (D1521, Promega, Madison, WI, USA). The DNA was fragmented by sonication to 100–600 bp with a Branson 450 Sonifier (Branson), followed by end repair with the End-It DNA End-Repair Kit (ER072, Epicentre). Paired-end cytosine-methylated adapters were ligated to the sonicated DNA for genomic DNA library construction. Adapter-ligated DNA of 200–400 bp was isolated by 2% agarose gel electrophoresis, and sodium bisulfite conversion was performed on it using the EZ DNA Methylation-Gold Kit (D5006, Zymo Research, Irvine, CA, USA) as per the manufacturer’s instructions. Half of the bisulfite-converted, adapter-ligated DNA molecules were enriched by 10 cycles of polymerase chain reaction (PCR) with the following reaction composition: 2.5 U of uracil-insensitive Pfu Turbo Cx Hotstart DNA Polymerase (600410, Stratagene), 5 μL 10× Pfu Turbo Reaction Buffer (600410, Stratagene), 25 mM deoxynucleotides (dNTPs), 0.5 μM TruSeq primer 1, and 0.5 μM TruSeq primer 2 (final volume 50 μL). The reaction products were purified using the MinElute PCR Purification Kit (28006, Qiagen, Hilden, Germany), separated by 2% agarose gel electrophoresis, and purified using the MinElute Gel Extraction Kit (28606, Qiagen). The bisulfite conversion rate was calculated as the percentage of cytosines sequenced at cytosine reference positions in the lambda genome. Libraries were sequenced on an Illumina HiSeq instrument (Illumina, San Diego, CA, USA) per the manufacturer’s instructions.

### ChIP-seq library generation and sequencing

ChIP was carried out as previously described with 20 μg chromatin (500 μg for EED) and 5 μg antibody with the following antibodies: EED (61203, Active Motif, Carlsbad, CA, USA), H3K4me3 (04-745, Millipore), H3K27ac (39133, Active Motif), and H3K27me3 (39155, Active Motif). ChIP and input library preparation and sequencing procedures were carried out as described previously [78].

### RNA-seq library generation and sequencing

Total RNA from WT and *Eed* ^-/-^ mESCs were extracted using Trizol (15596026, Ambion) according to the manufacturer’s instructions. We treated 10 μg RNA with DNase I (EN0521, Fermentas) at 37 °C for 1 h to remove DNA. Ribosomal RNA was removed using a Ribo-Zero Magnetic Gold Kit (MRZ11124C, Epicentre). Purified RNA was fragmented with RNA Fragmentation Buffer (E6186A, New England Biolabs (NEB), Ipswich, MA, USA) at 95 °C for 5 min and stopped with ethylenediaminetetraacetic acid (EDTA). The strand-specific RNA libraries were prepared as described previously [79] and sequenced on an Illumina HiSeq instrument per the manufacturer’s instructions.

### 4C-seq library generation and sequencing

Cells were crosslinked with 1% formaldehyde at room temperature for 10 min, then treated with 0.14 M glycine for 15 min. The crosslinked cells were incubated in lysis buffer (5 mM Tris at pH 7.5, 150 mM NaCl, 5 mM EDTA, 0.5% NP-40, 1% TritonX-100, and fresh proteinase inhibitor) for 1 h. After being centrifuged at 3000 rpm for 5 min, the supernatants were removed. The cell pellets were resuspended in 1× NEB buffer 2 with 50 U *Hin*dIII (NEB) and digested at 37 °C overnight. The digested samples were ligated in 1× T4 DNA ligase buffer with 2000 U T4 DNA ligase (NEB) at 24 °C for 6 h. The ligated products were treated with 5 μL Proteinase K (20 mg/mL) at 55 °C for 30 min, then incubated at 65 °C overnight. After reversing the crosslinking, the DNA was purified by phenol-chloroform extraction and precipitated with EtOH. The prepared DNA as a 3C library was used for 4C.

The 4C experiments were performed as described previously [80] with minor changes. Briefly, the 3C library was digested with 50 U Dpn II (NEB) at 37 °C overnight and ligated with 4000 U T4 DNA ligase (NEB) at 4 °C for 16 h. The DNA was purified by phenol-chloroform extraction and precipitated with EtOH. For each 4C library 200 ng of DNA was amplified with specific inverse primers using the Expand Long Range PCR System (Roche). First, 1.5 μM each of the short primers without TruSeq adapters were used to amplify the 4C libraries in a 25-μL reaction volume under the following program: 94 °C, 2 min; 10 cycles × (94 °C, 30 s; 55 °C, 1 min; 68 °C, 1 min); 68 °C, 7 min. PCR products were purified with AMPure beads to recover the DNA fragments of size 100—500 bp. The purified DNA products were amplified by the long primer pairs with the specific TruSeq adapters in a 50-μL volume as follows: 94 °C, 2 min; (94 °C, 30 s; 55 °C, 1 min; 68 °C, 1 min) × 10 cycles; 68 °C, 7 min; (94 °C, 30 s; 68 °C, 1 min + 20s/additional cycles; 68 °C, 1 min) × 15 cycles; 68 °C, 7 min. The final PCR products purified with AMPure beads were sequenced on an Illumina HiSeq instrument per the manufacturer’s instructions. The primers used in this study are listed in Table 1.

**Table 1:** Primers/oligos used in this study

*Tet1* sgRNA-F	CACCggctgctgtcagggagctca
*Tet1* sgRNA-R	AAACtgagctccctgacagcagcc
*Tet2* sgRNA-F	CACCgaaagtgccaacagatatcc
*Tet2* sgRNA-R	AAACggatatctgttggcactttc
*Tet3* sgRNA-F	CACCgagtgccccgacttcctcgag
*Tet3* sgRNA-R	AAACctcgaggaagtcggggcactc
*Eed* sgRNA 1-F	CACCgaggtgctgccgccttgtttt
*Eed* sgRNA 1-R	AAACaaaacaaggcggcagcacctc
*Eed* sgRNA 2-F	CACCgctacttttgaattcacatc
*Eed* sgRNA 2-R	AAACgatgtgaattcaaaagtagc
4C bait in DMV *Pax6*-F without adapters	tcagtgggagaggccactgg
4C bait in DMV *Pax6*-R without adapters	ccccacagtccatctctcag
4C bait in DMV *Pax6*-F with adapters	Aatgatacggcgaccaccgagatctacactctttccctacacgacg ctcttccgatcttcagtgggagaggccactgg
4C bait in DMV *Pax6*-R with adapters	Caagcagaagacggcatacgagatcaggatgtgactggagttcag acgtgtgctcttccg
4C bait out of DMV *Pax6*-F without adapters	aacagacattctttgccact
4C bait out of DMV *Pax6*-R without adapters	acatttggaggccacagatc
4C bait out of DMV *Pax6*-F with adapters	aatgatacggcgaccaccgagatctacactctttccctacacgacgctcttccgatctaacagacattctttgccact
4C bait out of DMV *Pax6*-R with adapters	caagcagaagacggcatacgagattgtggcgtgactggagttcagacgtgtgctcttccg
4C bait in DMV *Nkx2-2*-F without adapters	tccacgcagaattctttagt
4C bait in DMV *Nkx2-2*-R without adapters	tatctccagctgtgcctgt
4C bait in DMV *Nkx2-2*-F with adapters	aatgatacggcgaccaccgagatctacactctttccctacacgacgctcttccgatcttccacgcagaattctttagt
4C bait in DMV *Nkx2-2*-R with adapters	caagcagaagacggcatacgagattacgacgtgactggagttcagacgtgtgctcttccg
4C bait out of DMV *Nkx2-2-*F without adapters	gcctaggcactggaaaactg
4C bait out of DMV *Nkx2-2*-R without adapters	agaccaggactcacaccaca
4C bait out of DMV *Nkx2-2*-F with adapters	aatgatacggcgaccaccgagatctacactctttccctacacgacgctcttccgatctgcctaggcactggaaaactg
4C bait out of DMV *Nkx2-2*-R with adapters	caagcagaagacggcatacgagatacagacgtgactggagttcagacgtgtgctcttcc

### Western blot

Histone extracts were run on 10% sodium dodecyl sulfate polyacrylamide gel electrophoresis (SDS-PAGE) gel and transferred to a nitrocellulose membrane. The nitrocellulose membrane was blocked with 5% milk for 1 h and incubated with antibodies against H3K27me3 (39155, Active Motif), H3K4me3 (04-745, Millipore), or α-tubulin (BE0026, EasyBio) at 4 °C overnight. On the second day, the membrane was incubated with secondary antibody at room temperature for 1 h. Chemiluminescent detection was done using SuperSignal^TM^ West Dura Extended Duration Substrate (34076, Thermo Fisher).

### 5hmC dot blot

DNA samples were diluted to different concentration gradients and denatured with 0.4 M NaOH and 10 mM EDTA at 99 °C for 10 min. The denatured DNA was cooled on ice immediately and loaded to a nylon transfer membrane (RNP303B, GE) followed by UV crosslinking. The membrane was dried and blocked in 10% milk for 1 h. 5hmC antibody (39769, Active Motif) was 1:2000 diluted in 10% milk and incubated with the membrane at room temperature for 2 h. After being washed with Tris-buffered saline Tween 20 (TBST) five times, the membrane was incubated with secondary antibody at room temperature for 1 h. Chemiluminescent detection was done by SuperSignal West Dura Extended Duration Substrate (34076, Thermo).

### Data analyses

#### ChIP-seq data processing

For WT and *Eed* ^-/-^ mESCs, ChIP-seq reads were aligned to mm9 with Bowtie2 (version 2.2.2) with parameters -t -q -N 1 -L 25. All unmapped reads, multi-mapped reads, and PCR duplicates were removed. To generate the ChIP-seq signals for each histone modification shown in the University of California, Santa Cruz (UCSC) Genome Browser, we normalized the read counts by computing the number of reads per kilobase of bin per million reads sequenced (RPKM). To minimize the batch and cell type variation, the RPKM values were further normalized through Z-score transformation, by subtracting the mean of RPKM across the genome and dividing by the standard deviation of RPKM across the genome.

#### MethylC-seq data processing

For WT and mutant mESCs, MethylC-seq reads were aligned to mm9 using the Bisulfite Sequence Mapping Program (BSMAP) [81] with parameters –r 0 –w 100 –v 0.1 –A AGATCGGAAGAGC. Multi-mapped reads and PCR duplicates were removed. After mapping, those 200-bp bins with total CG coverage less than 5 were removed. Methylation level was calculated using methylated CpG versus total CpG in each bin.

#### RNA-seq data processing

For WT and *Eed* ^-/-^ mESCs, the RNA-seq reads were mapped to mm9 with TopHat (version 1.20). The mapped reads were further analyzed by Cufflinks [82], and the expression levels for each transcript were quantified as fragments per kilobase of transcript per million mapped reads (FPKM).

#### 4C-seq data processing

Reads with the 5’ end mapped to a 4C forward primer were selected from the total fastq file. The selected reads were mapped to the mm9 assembly with Bowtie2 (version 2.2.2). The mapped reads were further mapped to *Hin*dIII sites with the software fourSig [83]. The fragment counts were finally normalized per one million reads.

### Identification and classification of DMVs

DMVs were identified as previously described [14]. Briefly, the genome was first divided into 1-kb bins, and the DNA methylation level was averaged within each bin. Then a sliding 5-kb window (with 1-kb steps) was used to identify regions that have an averaged methylation level less than 0.15 in a 5-kb window. Continuous regions resulting from this analysis were then merged to form DMVs. Dynamic DMVs were defined as follows: all tsDMRs previously identified [15] with length over 2 kb were used in this analysis. DMVs with at least one entire tsDMR, or DMVs with more than half of the regions covered by tsDMRs, were defined as dynamic DMVs. Other DMVs were considered as constant DMVs (group I). Two additional groups of DMVs were identified from dynamic DMVs by analyzing the correlation between the changes of DNA methylation and transcription activities of nearby genes. Given DMVs frequently show similar patterns within each of the four lineages (blood, endoderm, mesoderm, and ectoderm as defined in [15]); we simplified the analysis by combining data from the same lineage. Specifically, for each DMV and related gene, the average DNA methylation levels and expression levels (FPKM) were computed for each of the four lineages (blood, endoderm, mesoderm, and ectoderm) [15]. We first identified dynamically regulated genes associated with DMVs. For a dynamically expressed gene, the FPKM in one lineage (highest among four lineages, with a minimal FPKM of 2) is three times more than the average FPKM in the other three lineages (with a maximal average FPKM of 2). For these dynamically expressed DMV genes, if we observed a negative correlation between expression and DNA methylation among the four lineages, the DMVs were clustered to group II. On the contrary, if we observed a positive correlation between expression and DNA methylation, those DMVs were clustered to group III.

## Additional files

Additional file 1: Figure S1. A global survey of DMVs in mouse somatic tissues. **Figure S2.** DMVs are hotspots of transcription factor binding sites and show low levels of deamination mutation rates. **Figure S3.** Identification of three groups of DMVs. **Figure S4.** Polycomb is required for maintenance of hypomethylation in DMVs. **Figure S5.** DMVs show a compact self-interacting structure. **Figure S6.** Polycomb regulates DMV hypomethylation likely through TETs. (PDF 3380 kb)

Additional file 2: Table S1. Summary of DMVs and associated genes for each mouse tissue and early developmental stage. (XLSX 666 kb)

Additional file 3: Table S2. List of orthologous DMV genes in human, mouse, and zebrafish. (XLS 26 kb)

## Abbreviations

4C: Circular chromosome conformation capture
ChIP: Chromatin immunoprecipitation
DMV: DNA methylation valley
DNMT: DNA methyltransferase
Eed: Embryonic ectoderm development
Ezh2: Enhancer of zeste homolog 2
HCGD: Hyper-conserved CpG island domains
MeDIP: Methylated DNA immunoprecipitation
MLL: Mixed-lineage leukemia
PGC: Primordial germ cell
PRC2: Polycomb repressive complex 2
TET: Ten-eleven translocation
TF: Transcription factor

## References

[1] Young RA (2011). Control of the embryonic stem cell state. Cell.

[2] Ng HH, Surani MA (2011). The transcriptional and signalling networks of pluripotency. Nat Cell Biol.

[3] Bonasio R, Tu S, Reinberg D (2010). Molecular signals of epigenetic states. Science.

[4] Reik W, Walter J (2001). Genomic imprinting: parental influence on the genome. Nat Rev Genet.

[5] Bartolomei MS, Ferguson-Smith AC (2011). Mammalian genomic imprinting. Cold Spring Harb Perspect Biol.

[6] Bird A (2002). DNA methylation patterns and epigenetic memory. Genes Dev.

[7] Li E, Bestor TH, Jaenisch R (1992). Targeted mutation of the DNA methyltransferase gene results in embryonic lethality. Cell.

[8] Okano M, Bell DW, Haber DA, Li E (1999). DNA methyltransferases Dnmt3a and Dnmt3b are essential for de novo methylation and mammalian development. Cell.

[9] Beard C, Li E, Jaenisch R (1995). Loss of methylation activates Xist in somatic but not in embryonic cells. Genes Dev.

[10] Vastenhouw NL, Schier AF (2012). Bivalent histone modifications in early embryogenesis. Curr Opin Cell Biol.

[11] Guenther MG, Levine SS, Boyer LA, Jaenisch R, Young RA (2007). A chromatin landmark and transcription initiation at most promoters in human cells. Cell.

[12] Simon JA, Kingston RE (2009). Mechanisms of polycomb gene silencing: knowns and unknowns. Nat Rev Mol Cell Biol.

[13] Lanzuolo C, Orlando V (2012). Memories from the Polycomb group proteins. Annu Rev Genet.

[14] Xie W, Schultz Matthew D, Lister R, Hou Z, Rajagopal N, Ray P, Whitaker John W, Tian S, Hawkins RD, Leung D (2013). Epigenomic analysis of multilineage differentiation of human embryonic stem cells. Cell.

[15] Hon GC, Rajagopal N, Shen Y, McCleary DF, Yue F, Dang MD, Ren B (2013). Epigenetic memory at embryonic enhancers identified in DNA methylation maps from adult mouse tissues. Nat Genet.

[16] Stadler MB, Murr R, Burger L, Ivanek R, Lienert F, Scholer A, van Nimwegen E, Wirbelauer C, Oakeley EJ, Gaidatzis D (2011). DNA-binding factors shape the mouse methylome at distal regulatory regions. Nature.

[17] Long HK, Sims D, Heger A, Blackledge NP, Kutter C, Wright ML, Grutzner F, Odom DT, Patient R, Ponting CP, Klose RJ (2013). Epigenetic conservation at gene regulatory elements revealed by non-methylated DNA profiling in seven vertebrates. Elife.

[18] Jeong M, Sun D, Luo M, Huang Y, Challen GA, Rodriguez B, Zhang X, Chavez L, Wang H, Hannah R (2014). Large conserved domains of low DNA methylation maintained by Dnmt3a. Nat Genet.

[19] Deaton AM, Bird A (2011). CpG islands and the regulation of transcription. Genes Dev.

[20] Boyer LA, Plath K, Zeitlinger J, Brambrink T, Medeiros LA, Lee TI, Levine SS, Wernig M, Tajonar A, Ray MK (2006). Polycomb complexes repress developmental regulators in murine embryonic stem cells. Nature.

[21] Bartke T, Vermeulen M, Xhemalce B, Robson SC, Mann M, Kouzarides T (2010). Nucleosome-interacting proteins regulated by DNA and histone methylation. Cell.

[22] Wu H, Coskun V, Tao J, Xie W, Ge W, Yoshikawa K, Li E, Zhang Y, Sun YE (2010). Dnmt3a-dependent nonpromoter DNA methylation facilitates transcription of neurogenic genes. Science.

[23] Brinkman AB, Gu H, Bartels SJ, Zhang Y, Matarese F, Simmer F, Marks H, Bock C, Gnirke A, Meissner A, Stunnenberg HG (2012). Sequential ChIP-bisulfite sequencing enables direct genome-scale investigation of chromatin and DNA methylation cross-talk. Genome Res.

[24] Reddington JP, Perricone SM, Nestor CE, Reichmann J, Youngson NA, Suzuki M, Reinhardt D, Dunican DS, Prendergast JG, Mjoseng H (2013). Redistribution of H3K27me3 upon DNA hypomethylation results in de-repression of Polycomb target genes. Genome Biol.

[25] Walter M, Teissandier A, Perez-Palacios R, Bourc’his D. An epigenetic switch ensures transposon repression upon dynamic loss of DNA methylation in embryonic stem cells. Elife. 2016;5. 10.7554/eLife.11418 PMC4769179 26814573

[26] Murphy PJ, Cipriany BR, Wallin CB, Ju CY, Szeto K, Hagarman JA, Benitez JJ, Craighead HG, Soloway PD (2013). Single-molecule analysis of combinatorial epigenomic states in normal and tumor cells. Proc Natl Acad Sci U S A.

[27] Statham AL, Robinson MD, Song JZ, Coolen MW, Stirzaker C, Clark SJ (2012). Bisulfite sequencing of chromatin immunoprecipitated DNA (BisChIP-seq) directly informs methylation status of histone-modified DNA. Genome Res.

[28] Hagarman JA, Motley MP, Kristjansdottir K, Soloway PD (2013). Coordinate regulation of DNA methylation and H3K27me3 in mouse embryonic stem cells. PLoS One.

[29] Harris RA, Wang T, Coarfa C, Nagarajan RP, Hong C, Downey SL, Johnson BE, Fouse SD, Delaney A, Zhao Y (2010). Comparison of sequencing-based methods to profile DNA methylation and identification of monoallelic epigenetic modifications. Nat Biotechnol.

[30] Kobayashi H, Sakurai T, Miura F, Imai M, Mochiduki K, Yanagisawa E, Sakashita A, Wakai T, Suzuki Y, Ito T (2013). High-resolution DNA methylome analysis of primordial germ cells identifies gender-specific reprogramming in mice. Genome Res.

[31] Wang L, Zhang J, Duan J, Gao X, Zhu W, Lu X, Yang L, Zhang J, Li G, Ci W (2014). Programming and inheritance of parental DNA methylomes in mammals. Cell.

[32] Zhang Y, Xiang Y, Yin Q, Du Z, Peng X, Wang Q, Fidalgo M, Xia W, Li Y, Zhao ZA (2018). Dynamic epigenomic landscapes during early lineage specification in mouse embryos. Nat Genet.

[33] Potok ME, Nix DA, Parnell TJ, Cairns BR (2013). Reprogramming the maternal zebrafish genome after fertilization to match the paternal methylation pattern. Cell.

[34] Gerstein MB, Kundaje A, Hariharan M, Landt SG, Yan KK, Cheng C, Mu XJ, Khurana E, Rozowsky J, Alexander R (2012). Architecture of the human regulatory network derived from ENCODE data. Nature.

[35] Wang J, Zhuang J, Iyer S, Lin X, Whitfield TW, Greven MC, Pierce BG, Dong X, Kundaje A, Cheng Y (2012). Sequence features and chromatin structure around the genomic regions bound by 119 human transcription factors. Genome Res.

[36] Wang J, Zhuang J, Iyer S, Lin XY, Greven MC, Kim BH, Moore J, Pierce BG, Dong X, Virgil D (2013). Factorbook.org: a Wiki-based database for transcription factor-binding data generated by the ENCODE consortium. Nucleic Acids Res.

[37] Hnisz D, Abraham BJ, Lee TI, Lau A, Saint-Andre V, Sigova AA, Hoke HA, Young RA (2013). Super-enhancers in the control of cell identity and disease. Cell.

[38] Chen X, Xu H, Yuan P, Fang F, Huss M, Vega VB, Wong E, Orlov YL, Zhang W, Jiang J (2008). Integration of external signaling pathways with the core transcriptional network in embryonic stem cells. Cell.

[39] Lee CK, Shibata Y, Rao B, Strahl BD, Lieb JD (2004). Evidence for nucleosome depletion at active regulatory regions genome-wide. Nat Genet.

[40] Alexandrov LB, Nik-Zainal S, Wedge DC, Aparicio SA, Behjati S, Biankin AV, Bignell GR, Bolli N, Borg A, Borresen-Dale AL (2013). Signatures of mutational processes in human cancer. Nature.

[41] Tanay A, O’Donnell AH, Damelin M, Bestor TH (2007). Hyperconserved CpG domains underlie Polycomb-binding sites. Proc Natl Acad Sci U S A.

[42] Cohen NM, Kenigsberg E, Tanay A (2011). Primate CpG islands are maintained by heterogeneous evolutionary regimes involving minimal selection. Cell.

[43] Shen Y, Yue F, McCleary DF, Ye Z, Edsall L, Kuan S, Wagner U, Dixon J, Lee L, Lobanenkov VV, Ren B (2012). A map of the cis-regulatory sequences in the mouse genome. Nature.

[44] Whyte Warren A, Orlando David A, Hnisz D, Abraham Brian J, Lin Charles Y, Kagey Michael H, Rahl Peter B, Lee Tong I, Young RA (2013). Master transcription factors and mediator establish super-enhancers at key cell identity genes. Cell.

[45] Ku M, Koche RP, Rheinbay E, Mendenhall EM, Endoh M, Mikkelsen TS, Presser A, Nusbaum C, Xie X, Chi AS (2008). Genomewide analysis of PRC1 and PRC2 occupancy identifies two classes of bivalent domains. PLoS Genet.

[46] Montgomery ND, Yee D, Chen A, Kalantry S, Chamberlain SJ, Otte AP, Magnuson T (2005). The murine polycomb group protein Eed is required for global histone H3 lysine-27 methylation. Curr Biol.

[47] Jones PA (2012). Functions of DNA methylation: islands, start sites, gene bodies and beyond. Nat Rev Genet.

[48] Baubec T, Colombo DF, Wirbelauer C, Schmidt J, Burger L, Krebs AR, Akalin A, Schubeler D (2015). Genomic profiling of DNA methyltransferases reveals a role for DNMT3B in genic methylation. Nature.

[49] Dhayalan A, Rajavelu A, Rathert P, Tamas R, Jurkowska RZ, Ragozin S, Jeltsch A (2010). The Dnmt3a PWWP domain reads histone 3 lysine 36 trimethylation and guides DNA methylation. J Biol Chem.

[50] Riising EM, Comet I, Leblanc B, Wu X, Johansen JV, Helin K (2014). Gene silencing triggers polycomb repressive complex 2 recruitment to CpG islands genome wide. Mol Cell.

[51] Ferrari KJ, Scelfo A, Jammula S, Cuomo A, Barozzi I, Stutzer A, Fischle W, Bonaldi T, Pasini D (2014). Polycomb-dependent H3K27me1 and H3K27me2 regulate active transcription and enhancer fidelity. Mol Cell.

[52] Margueron R, Li G, Sarma K, Blais A, Zavadil J, Woodcock CL, Dynlacht BD, Reinberg D (2008). Ezh1 and Ezh2 maintain repressive chromatin through different mechanisms. Mol Cell.

[53] Eskeland R, Leeb M, Grimes GR, Kress C, Boyle S, Sproul D, Gilbert N, Fan Y, Skoultchi AI, Wutz A, Bickmore WA (2010). Ring1B compacts chromatin structure and represses gene expression independent of histone ubiquitination. Mol Cell.

[54] van der Vlag J, Otte AP (1999). Transcriptional repression mediated by the human polycomb-group protein EED involves histone deacetylation. Nat Genet.

[55] Pasini D, Malatesta M, Jung HR, Walfridsson J, Willer A, Olsson L, Skotte J, Wutz A, Porse B, Jensen ON, Helin K (2010). Characterization of an antagonistic switch between histone H3 lysine 27 methylation and acetylation in the transcriptional regulation of Polycomb group target genes. Nucleic Acids Res.

[56] Ai S, Peng Y, Li C, Gu F, Yu X, Yue Y, Ma Q, Chen J, Lin Z, Zhou P (2017). EED orchestration of heart maturation through interaction with HDACs is H3K27me3-independent. Elife.

[57] Eskeland R, Freyer E, Leeb M, Wutz A, Bickmore WA (2010). Histone acetylation and the maintenance of chromatin compaction by Polycomb repressive complexes. Cold Spring Harb Symp Quant Biol.

[58] Kundu S, Ji F, Sunwoo H, Jain G, Lee JT, Sadreyev RI, Dekker J, Kingston RE (2017). Polycomb repressive complex 1 generates discrete compacted domains that change during differentiation. Mol Cell.

[59] Denholtz M, Bonora G, Chronis C, Splinter E, de Laat W, Ernst J, Pellegrini M, Plath K (2013). Long-range chromatin contacts in embryonic stem cells reveal a role for pluripotency factors and polycomb proteins in genome organization. Cell Stem Cell.

[60] Joshi O, Wang SY, Kuznetsova T, Atlasi Y, Peng T, Fabre PJ, Habibi E, Shaik J, Saeed S, Handoko L (2015). Dynamic reorganization of extremely long-range promoter-promoter interactions between two states of pluripotency. Cell Stem Cell.

[61] Schoenfelder S, Sugar R, Dimond A, Javierre BM, Armstrong H, Mifsud B, Dimitrova E, Matheson L, Tavares-Cadete F, Furlan-Magaril M (2015). Polycomb repressive complex PRC1 spatially constrains the mouse embryonic stem cell genome. Nat Genet.

[62] Vieux-Rochas M, Fabre PJ, Leleu M, Duboule D, Noordermeer D (2015). Clustering of mammalian Hox genes with other H3K27me3 targets within an active nuclear domain. Proc Natl Acad Sci U S A.

[63] Verma N, Pan H, Dore LC, Shukla A, Li QV, Pelham-Webb B, Teijeiro V, Gonzalez F, Krivtsov A, Chang CJ (2018). TET proteins safeguard bivalent promoters from de novo methylation in human embryonic stem cells. Nat Genet.

[64] Wiehle L, Raddatz G, Musch T, Dawlaty MM, Jaenisch R, Lyko F, Breiling A (2015). Tet1 and Tet2 protect DNA methylation canyons against hypermethylation. Mol Cell Biol.

[65] Neri F, Incarnato D, Krepelova A, Rapelli S, Pagnani A, Zecchina R, Parlato C, Oliviero S (2013). Genome-wide analysis identifies a functional association of Tet1 and Polycomb repressive complex 2 in mouse embryonic stem cells. Genome Biol.

[66] Wu H, D’Alessio AC, Ito S, Xia K, Wang Z, Cui K, Zhao K, Sun YE, Zhang Y (2011). Dual functions of Tet1 in transcriptional regulation in mouse embryonic stem cells. Nature.

[67] Lu F, Liu Y, Jiang L, Yamaguchi S, Zhang Y (2014). Role of Tet proteins in enhancer activity and telomere elongation. Genes Dev.

[68] King AD, Huang K, Rubbi L, Liu S, Wang CY, Wang Y, Pellegrini M, Fan G (2016). Reversible regulation of promoter and enhancer histone landscape by DNA methylation in mouse embryonic stem cells. Cell Rep.

[69] Schlesinger Y, Straussman R, Keshet I, Farkash S, Hecht M, Zimmerman J, Eden E, Yakhini Z, Ben-Shushan E, Reubinoff BE (2007). Polycomb-mediated methylation on Lys27 of histone H3 pre-marks genes for de novo methylation in cancer. Nat Genet.

[70] Easwaran H, Johnstone SE, Van Neste L, Ohm J, Mosbruger T, Wang Q, Aryee MJ, Joyce P, Ahuja N, Weisenberger D (2012). A DNA hypermethylation module for the stem/progenitor cell signature of cancer. Genome Res.

[71] Ohm JE, McGarvey KM, Yu X, Cheng L, Schuebel KE, Cope L, Mohammad HP, Chen W, Daniel VC, Yu W (2007). A stem cell-like chromatin pattern may predispose tumor suppressor genes to DNA hypermethylation and heritable silencing. Nat Genet.

[72] Widschwendter M, Fiegl H, Egle D, Mueller-Holzner E, Spizzo G, Marth C, Weisenberger DJ, Campan M, Young J, Jacobs I, Laird PW (2007). Epigenetic stem cell signature in cancer. Nat Genet.

[73] Sproul D, Meehan RR (2013). Genomic insights into cancer-associated aberrant CpG island hypermethylation. Brief Funct Genomics.

[74] Varambally S, Dhanasekaran SM, Zhou M, Barrette TR, Kumar-Sinha C, Sanda MG, Ghosh D, Pienta KJ, Sewalt RG, Otte AP (2002). The polycomb group protein EZH2 is involved in progression of prostate cancer. Nature.

[75] McGarvey KM, Greene E, Fahrner JA, Jenuwein T, Baylin SB (2007). DNA methylation and complete transcriptional silencing of cancer genes persist after depletion of EZH2. Cancer Res.

[76] Branciamore S, Chen ZX, Riggs AD, Rodin SN (2010). CpG island clusters and pro-epigenetic selection for CpGs in protein-coding exons of HOX and other transcription factors. Proc Natl Acad Sci U S A.

[77] Ran FA, Hsu PD, Wright J, Agarwala V, Scott DA, Zhang F (2013). Genome engineering using the CRISPR-Cas9 system. Nat Protoc.

[78] Hawkins RD, Hon GC, Lee LK, Ngo Q, Lister R, Pelizzola M, Edsall LE, Kuan S, Luu Y, Klugman S (2010). Distinct epigenomic landscapes of pluripotent and lineage-committed human cells. Cell Stem Cell.

[79] Parkhomchuk D, Borodina T, Amstislavskiy V, Banaru M, Hallen L, Krobitsch S, Lehrach H, Soldatov A (2009). Transcriptome analysis by strand-specific sequencing of complementary DNA. Nucleic Acids Res.

[80] van de Werken HJ, de Vree PJ, Splinter E, Holwerda SJ, Klous P, de Wit E, de Laat W (2012). 4C technology: protocols and data analysis. Methods Enzymol.

[81] Xi Y, Li W (2009). BSMAP: whole genome bisulfite sequence MAPping program. BMC Bioinf.

[82] Trapnell C, Roberts A, Goff L, Pertea G, Kim D, Kelley DR, Pimentel H, Salzberg SL, Rinn JL, Pachter L (2012). Differential gene and transcript expression analysis of RNA-seq experiments with TopHat and Cufflinks. Nat Protoc.

[83] Williams RL, Starmer J, Mugford JW, Calabrese JM, Mieczkowski P, Yee D, Magnuson T (2014). fourSig: a method for determining chromosomal interactions in 4C-Seq data. Nucleic Acids Res.

[84] Li Y, Zheng H, Wang Q, Zhou C, Wei L, Liu X, Zhang W, Zhang Y, Du Z, Wang X, Xie W: Genomewide analyses reveal a role of Polycomb in maintaining hypomethylation of DNA methylation valley. https://www.ncbi.nlm.nih.gov/geo/query/acc.cgi?acc=GSE102753. 10.1186/s13059-018-1390-8 PMC5806489 29422066

[85] Molaro A, Hodges E, Fang F, Song Q, McCombie WR, Hannon GJ, Smith AD (2011). Sperm methylation profiles reveal features of epigenetic inheritance and evolution in primates. Cell.

