# Supplementary material for: Genome-wide analyses reveal a role of Polycomb in promoting hypomethylation of DNA methylation valleys
Source: Genome Biol. 2018 Feb 8;19:18. doi: 10.1186/s13059-018-1390-8 (PMC5806489; doi:10.1186/s13059-018-1390-8)
Supplement: Supplementary file 1 — A global survey of DMVs in mouse somatic tissues. Figure S2. DMVs are hotspots of transcription factor binding sites and show low levels of deamination mutation rates. Figure S3. Identification of three groups of DMVs. Figure S4. Polycomb is required for maintenance of hypomethylation in DMVs. Figure S5. DMVs show a compact self-interacting structure. Figure S6. Polycomb regulates DMV hypomethylation likely through TETs. (PDF 3380 kb) [file 13059_2018_1390_MOESM1_ESM.pdf]

Supplementary Figure Legends

Figure S1. A global survey of DMVs in mouse somatic tissues.

(A) UCSC Genome Browser snapshot showing DNA methylomes near a DMV (*Foxd3*) in mouse somatic tissues [1].

(B) The numbers of DMVs found in different mouse cell types shown as bar graphs.

(C) Maternal and paternal DNA methylation patterns in different regions (promoter, gene body, intergenic, CGI and tissue DMV) during mouse development.

Figure S2. DMVs are hotspots of transcription factor binding sites and show low levels of deamination mutation rates.

(A) TF binding density (number of TF binding sites per Kb) in CpG island (CGI) and non-CpG island (non-CGI) of DMVs using a ChIP-seq dataset from ENCODE for a panel of TFs [2-4]. A random set of regions with the same length of each DMV were similarly analyzed as random control.

(B) TF binding site distribution around the TSSs (transcription start sites) in mouse for promoters in DMVs or other promoters using a published ChIP-seq dataset [5].

(C) The average DNA methylation level and nucleosome occupancy enrichment in mESC [6] are shown around DMVs.

(D) Venn diagram shows the overlap between CGI clusters and DMVs (left top). Boxplot showing PhastCons scores for CGI clusters that overlap with DMVs or do not overlap with DMVs (left bottom). Similar comparison analysis results are also shown for super-enhancers and DMVs (right). CGI clusters [7] and super-enhancers in human embryonic stem cells [8] were defined previously.

(E) UCSC Genome Browser snapshot showing the sequence deamination rate in the

human genome [9], DNA methylomes for various human cell lines [10], and CG density for a DMV (*Pax6*).

(F) Boxplots showing the deamination rates for non-CGI regions (near promoters, TSS±10kb) with different CG densities that either fall inside or outside DMVs. Non-CGI regions near promoters were chosen for better comparison as non-CGI regions away from promoters show even higher deamination rates (data not shown).

Figure S3. Identification of three groups of DMVs.

(A) Hierarchical clustering analysis for mouse tissues based on their DMV DNA methylation levels [1].

(B) Boxplots showing the length (left), CG density (middle), and GC content (right) of group I (yellow), group II (blue) and group III (red) DMVs.

(C) Gene ontology analysis results for genes associated with group I, group II and group III DMVs.

Figure S4. Polycomb is required for maintenance of hypomethylation in DMVs.

(A) Boxplots show enrichment of H3K27me3 and Polycomb protein in highly DNA methylated and lowly DNA methylated H3K27me3 peaks. The regions that are depleted of H3K27me3 and Polycomb bindings (green) were selected as negative control.

(B) Western blot of H3K27me3 in WT and *Eed* $^{-/-}$ mESCs. H3K4me3 is used as control.

(C) Heatmaps representing the difference of DNA methylation levels within DMVs between *Eed* $^{-/-}$ mESCs and multiple WT mESCs with various background,

including TT2 [7], R1, and 159-2 [11]. All DMVs are normalized to the same length. The levels of H3K27me3 in WT mESCs are also shown.

(D) Bar graphs showing the FPKM (fragments per kilobase per million mapped reads) of pluripotency genes and Polycomb target genes in WT (blue) and *Eed* $^{-/-}$ (red) mESCs. A zoomed-in graph is also shown.

(E) Boxplot showing difference of gene expression (FPKM) between *Eed*$^{-/-}$ and WT in hypermethylated and unchanged DMVs. All Polycomb DMVs are sorted according to the difference of DNA methylation between *Eed*$^{-/-}$ and WT. The top 100 and bottom 100 of DMVs are defined as hypermethylated and unchanged, respectively.

(F) The epigenetic landscape is shown for a DMV gene (*Pou3f2*) marked by both H3K27me3 and Polycomb, and a gene (*Gm6116*) marked only by H3K27me3. Regions with elevated DNA methylation in *Eed* $^{-/-}$ mESCs compared to WT are shaded. Binding of EED, EZH2, and RING1B [12] is also shown.

Figure S5. DMVs show a compact self-interacting structure.

(A) Snapshot showing 4C-seq results for DMV regions *Pax6* and *Nkx2-2* in WT and *Eed* $^{-/-}$ mESCs (with two replicates shown). Two distal regions (shaded by red) that also interact with DMVs (shaded by purple) are shown on the right (*Wt1* and *Foxa2*).

(B) Snapshot showing 4C-seq results for the HoxB region in WT and *Eed* $^{-/-}$ mESCs using a bait designed for the *Hoxb3* gene. DNA methylation and H3K27me3 in mESCs are also shown. The 4C-seq data from Denholtz et al. [13] is used as positive control.

Figure S6. Polycomb regulates DMV hypomethylation likely through TETs.

(A) Boxplots showing the DNA methylation change (KO-WT) of Polycomb DMVs and other DMVs for mESCs deficient for *Eed* or *Tet1/2/3* [14]. P-values (two-sided t-test) are also shown.

(B) Boxplots showing the DNA methylation change (KO-WT) of CpG islands and non-CpG island regions within DMVs for mESCs deficient for *Eed* or *Tet1/2/3* [14]. P-values (two-sided t-test) are also shown.

(C) Dot blot assay results for 5hmC in WT, *Tet* TKO and *Tet/Eed* QKO mESCs.

(D) Western blot of H3K27me3 in *Tet* TKO and *Tet/Eed* QKO mESCs. α bulin is used as control.

(E) The average enrichment of EED around Polycomb DMVs in WT and *Dnmt3a/3b* DKO mESCs.

Reference:

1. Hon GC, Rajagopal N, Shen Y, McCleary DF, Yue F, Dang MD, Ren B: **Epigenetic memory at embryonic enhancers identified in DNA methylation maps from adult mouse tissues.** *Nat Genet* 2013, **45:**1198-1206.
2. Gerstein MB, Kundaje A, Hariharan M, Landt SG, Yan KK, Cheng C, Mu XJ, Khurana E, Rozowsky J, Alexander R, et al: **Architecture of the human regulatory network derived from ENCODE data.** *Nature* 2012, **489:**91-100.
3. Wang J, Zhuang J, Iyer S, Lin X, Whitfield TW, Greven MC, Pierce BG, Dong X, Kundaje A, Cheng Y, et al: **Sequence features and chromatin structure around the genomic regions bound by 119 human transcription factors.** *Genome Res* 2012, **22:**1798-1812.
4. Wang J, Zhuang J, Iyer S, Lin XY, Greven MC, Kim BH, Moore J, Pierce BG, Dong X, Virgil D, et al: **Factorbook.org: a Wiki-based database for

transcription factor-binding data generated by the ENCODE consortium. *Nucleic Acids Res* 2013, **41:**D171-176.

5. Chen X, Xu H, Yuan P, Fang F, Huss M, Vega VB, Wong E, Orlov YL, Zhang W, Jiang J, et al: **Integration of external signaling pathways with the core transcriptional network in embryonic stem cells.** *Cell* 2008, **133:**1106-1117.
6. Teif VB, Vainshtein Y, Caudron-Herger M, Mallm JP, Marth C, Hofer T, Rippe K: **Genome-wide nucleosome positioning during embryonic stem cell development.** *Nat Struct Mol Biol* 2012, **19:**1185-1192.
7. Xie W, Schultz Matthew D, Lister R, Hou Z, Rajagopal N, Ray P, Whitaker John W, Tian S, Hawkins RD, Leung D, et al: **Epigenomic Analysis of Multilineage Differentiation of Human Embryonic Stem Cells.** *Cell* 2013, **153:**1134-1148.
8. Hnisz D, Abraham BJ, Lee TI, Lau A, Saint-Andre V, Sigova AA, Hoke HA, Young RA: **Super-enhancers in the control of cell identity and disease.** *Cell* 2013, **155:**934-947.
9. Cohen NM, Kenigsberg E, Tanay A: **Primate CpG islands are maintained by heterogeneous evolutionary regimes involving minimal selection.** *Cell* 2011, **145:**773-786.
10. Xie W, Schultz MD, Lister R, Hou Z, Rajagopal N, Ray P, Whitaker JW, Tian S, Hawkins RD, Leung D, et al: **Epigenomic analysis of multilineage differentiation of human embryonic stem cells.** *Cell* 2013, **153:**1134-1148.
11. Stadler MB, Murr R, Burger L, Ivanek R, Lienert F, Scholer A, van Nimwegen E, Wirbelauer C, Oakeley EJ, Gaidatzis D, et al: **DNA-binding factors shape the mouse methylome at distal regulatory regions.** *Nature* 2011, **480:**490-495.
12. Ku M, Koche RP, Rheinbay E, Mendenhall EM, Endoh M, Mikkelsen TS, Presser A, Nusbaum C, Xie X, Chi AS, et al: **Genomewide analysis of PRC1 and PRC2 occupancy identifies two classes of bivalent domains.** *PLoS Genet* 2008, **4:**e1000242.
13. Denholtz M, Bonora G, Chronis C, Splinter E, de Laat W, Ernst J, Pellegrini M, Plath K: **Long-range chromatin contacts in embryonic stem cells reveal a role for pluripotency factors and polycomb proteins in genome organization.** *Cell Stem Cell* 2013, **13:**602-616.

14. Lu F, Liu Y, Jiang L, Yamaguchi S, Zhang Y: **Role of Tet proteins in enhancer activity and telomere elongation.** *Genes Dev* 2014, **28:**2103-2119.

Figure S1

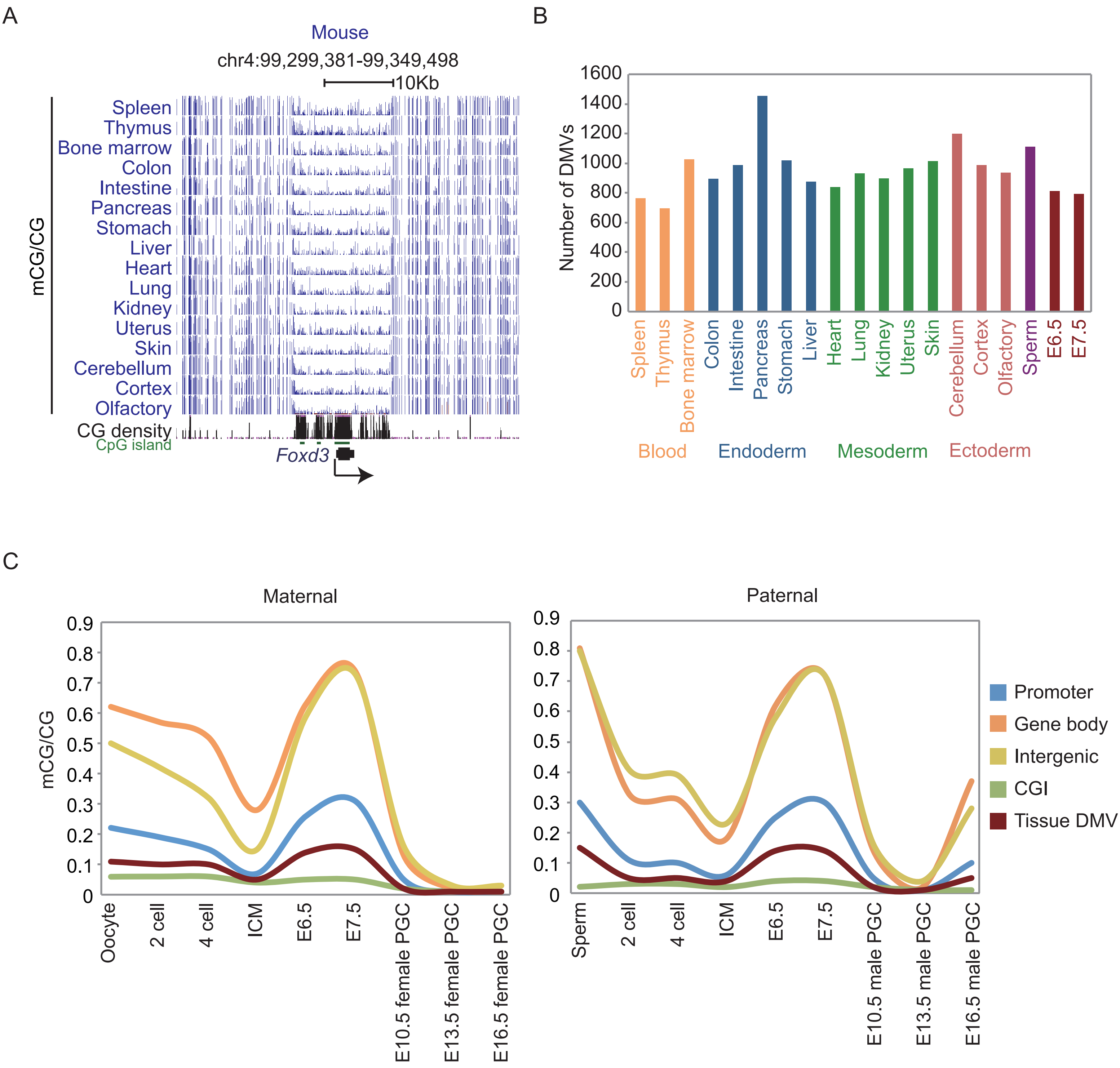
A
Mouse
chr4:99,299,381-99,349,498
10Kb
mCG/CG
Spleen
Thymus
Bone marrow
Colon
Intestine
Pancreas
Stomach
Liver
Heart
Lung
Kidney
Uterus
Skin
Cerebellum
Cortex
Olfactory
CG density
CpG island
Foxd3
B
Number of DMVs
1600
1400
1200
1000
800
600
400
200
0
Spleen
Thymus
Bone marrow
Colon
Intestine
Pancreas
Stomach
Liver
Heart
Lung
Kidney
Uterus
Skin
Cerebellum
Cortex
Olfactory
Sperm
E6.5
E7.5
Blood
Endoderm
Mesoderm
Ectoderm
C
Maternal
Paternal
mCG/CG
0.9
0.8
0.7
0.6
0.5
0.4
0.3
0.2
0.1
0
Oocyte
2 cell
4 cell
ICM
E6.5
E7.5
E10.5 female PGC
E13.5 female PGC
E16.5 female PGC
Sperm
E10.5 male PGC
E13.5 male PGC
E16.5 male PGC
Promoter
Gene body
Intergenic
CGI
Tissue DMV

Figure S2

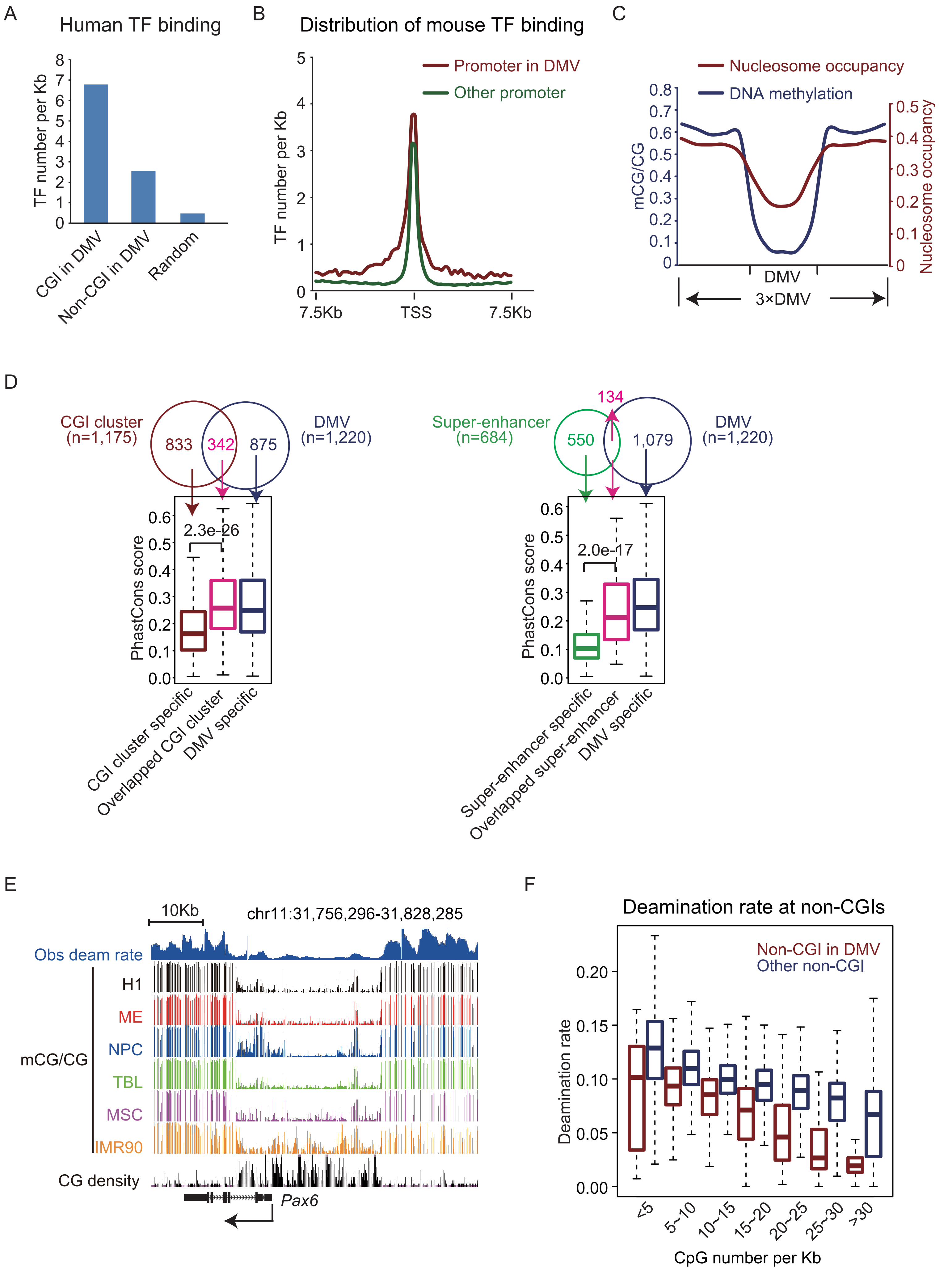
A
Human TF binding
TF number per Kb
CGI in DMV
Non-CGI in DMV
Random
B
Distribution of mouse TF binding
Promoter in DMV
Other promoter
TF number per Kb
7.5Kb
TSS
7.5Kb
C
Nucleosome occupancy
DNA methylation
mCG/CG
Nucleosome occupancy
DMV
3×DMV
D
CGI cluster
(n=1,175)
833
342
875
DMV
(n=1,220)
PhastCons score
2.3e-26
CGI cluster specific
Overlapped CGI cluster
DMV specific
Super-enhancer
(n=684)
134
550
1,079
DMV
(n=1,220)
PhastCons score
2.0e-17
Super-enhancer specific
Overlapped super-enhancer
DMV specific
E
10Kb
chr11:31,756,296-31,828,285
Obs deam rate
H1
ME
NPC
TBL
MSC
IMR90
mCG/CG
CG density
Pax6
F
Deamination rate at non-CGIs
Non-CGI in DMV
Other non-CGI
Deamination rate
<5
5~10
10~15
15~20
20~25
25~30
>30
CpG number per Kb

Figure S3

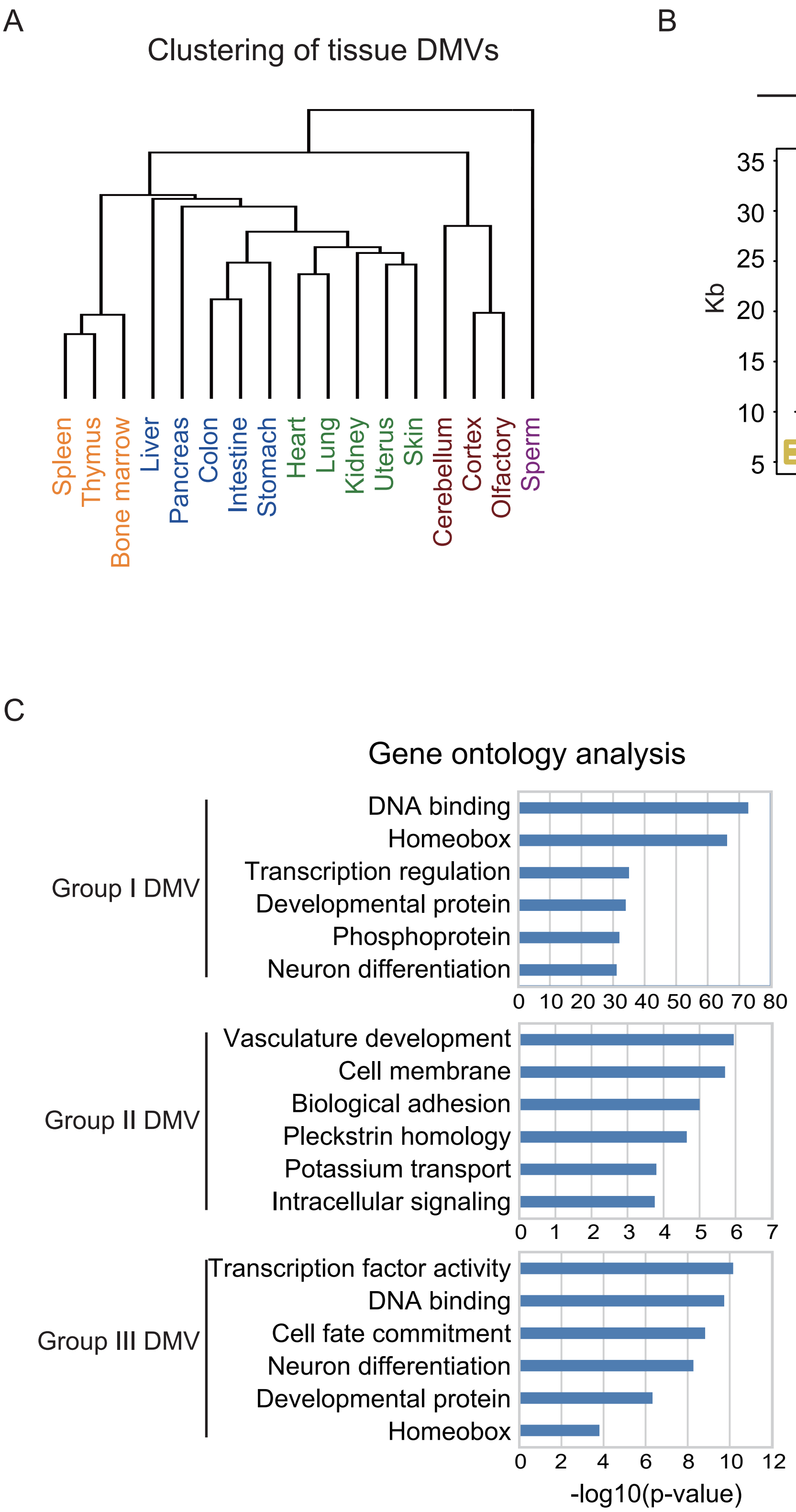
A
Clustering of tissue DMVs
Spleen
Thymus
Bone marrow
Liver
Pancreas
Colon
Intestine
Stomach
Heart
Lung
Kidney
Uterus
Skin
Cerebellum
Cortex
Olfactory
Sperm
B
DMV
Length
CG density
GC content
Kb
35
30
25
20
15
10
5
CpG number per Kb
60
50
40
30
20
10
Percentage
65%
60%
55%
50%
45%
40%
35%
Group I
Group II
Group III
C
Gene ontology analysis
Group I DMV
DNA binding
Homeobox
Transcription regulation
Developmental protein
Phosphoprotein
Neuron differentiation
0 10 20 30 40 50 60 70 80
Group II DMV
Vasculature development
Cell membrane
Biological adhesion
Pleckstrin homology
Potassium transport
Intracellular signaling
0 1 2 3 4 5 6 7
Group III DMV
Transcription factor activity
DNA binding
Cell fate commitment
Neuron differentiation
Developmental protein
Homeobox
0 2 4 6 8 10 12
-log10(p-value)

Figure S4

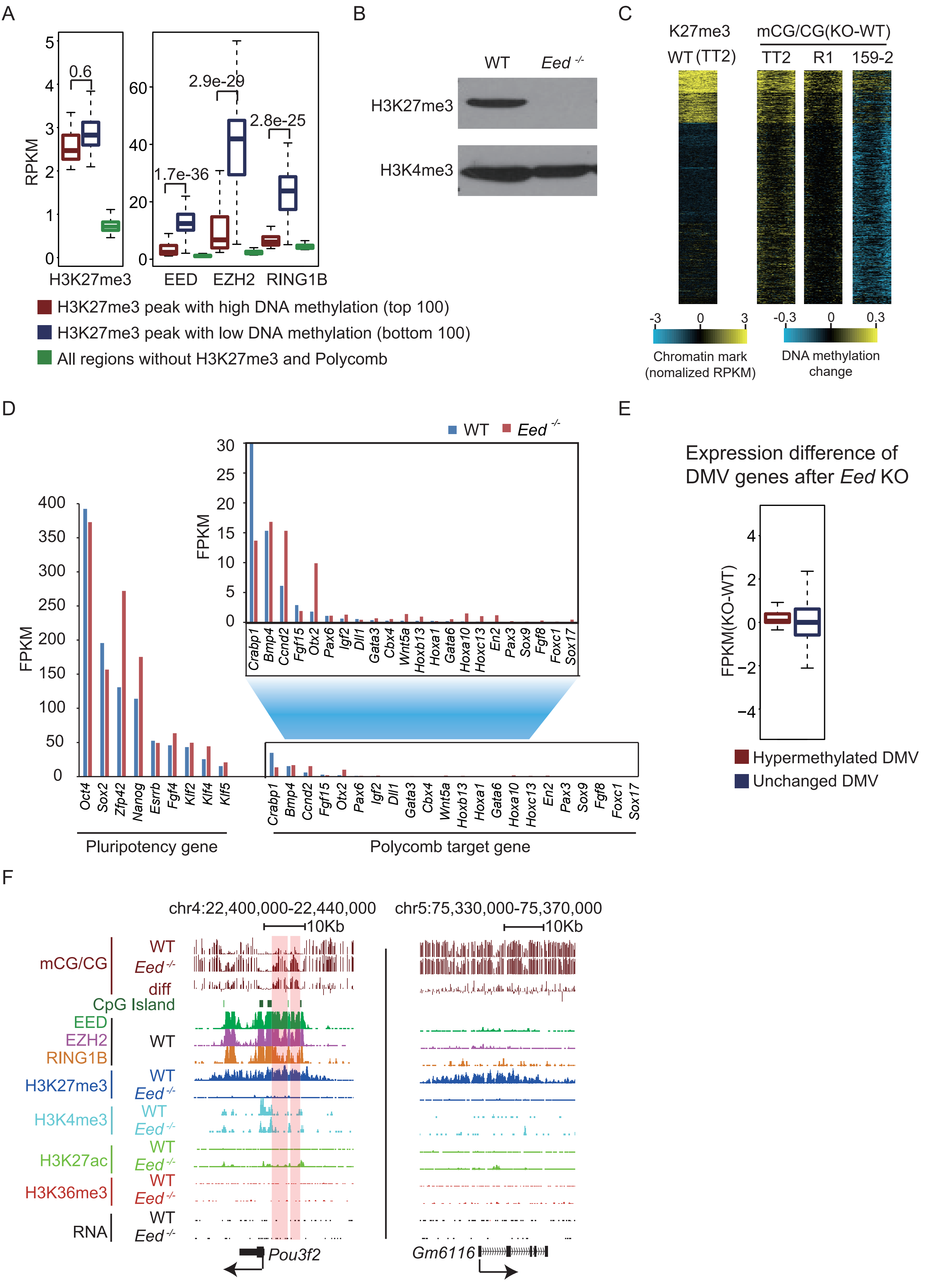

A
RPKM
0.6
2.9e-29
2.8e-25
1.7e-36
H3K27me3
EED
EZH2
RING1B
H3K27me3 peak with high DNA methylation (top 100)
H3K27me3 peak with low DNA methylation (bottom 100)
All regions without H3K27me3 and Polycomb
B
WT
Eed -/-
H3K27me3
H3K4me3
C
K27me3
WT (TT2)
mCG/CG(KO-WT)
TT2
R1
159-2
Chromatin mark (nomalized RPKM)
DNA methylation change
D
WT
Eed -/-
FPKM
Oct4
Sox2
Zfp42
Nanog
Esrrb
Fgf4
Klf2
Klf4
Klf5
Crabp1
Bmp4
Ccnd2
Fgf15
Otx2
Pax6
Igf2
Dll1
Gata3
Cbx4
Wnt5a
Hoxb13
Hoxa1
Gata6
Hoxa10
Hoxc13
En2
Pax3
Sox9
Fgf8
Foxc1
Sox17
Pluripotency gene
Polycomb target gene
E
Expression difference of DMV genes after Eed KO
FPKM(KO-WT)
Hypermethylated DMV
Unchanged DMV
F
chr4:22,400,000-22,440,000
chr5:75,330,000-75,370,000
10Kb
mCG/CG
WT
Eed -/-
diff
CpG Island
EED
EZH2
RING1B
H3K27me3
H3K4me3
H3K27ac
H3K36me3
RNA
Pou3f2
Gm6116

Figure S5

A

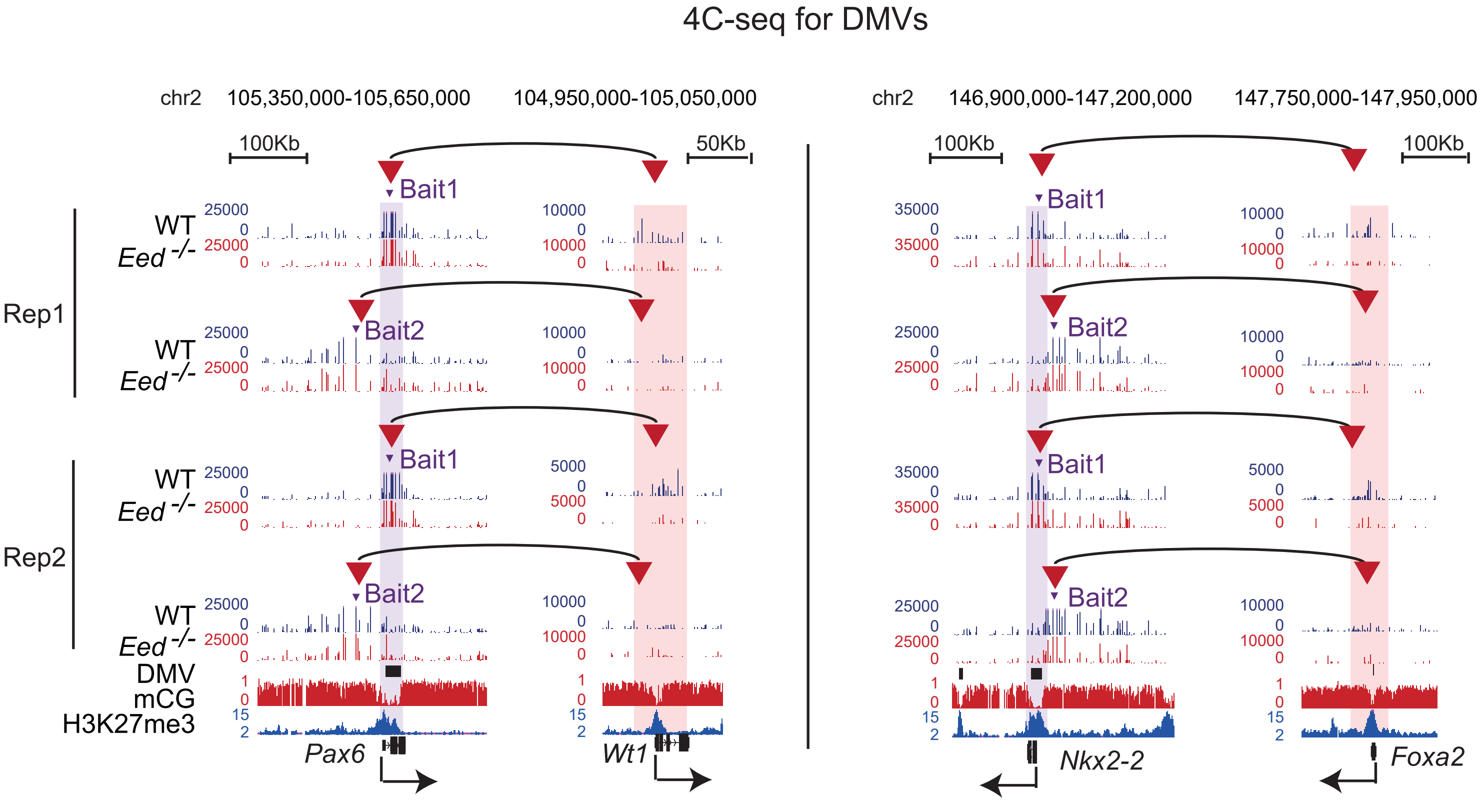
4C-seq for DMVs
chr2
105,350,000-105,650,000
104,950,000-105,050,000
146,900,000-147,200,000
147,750,000-147,950,000
100Kb
50Kb
Bait1
Bait2
WT
Eed -/-
Rep1
Rep2
DMV
mCG
H3K27me3
Pax6
Wt1
Nkx2-2
Foxa2

B

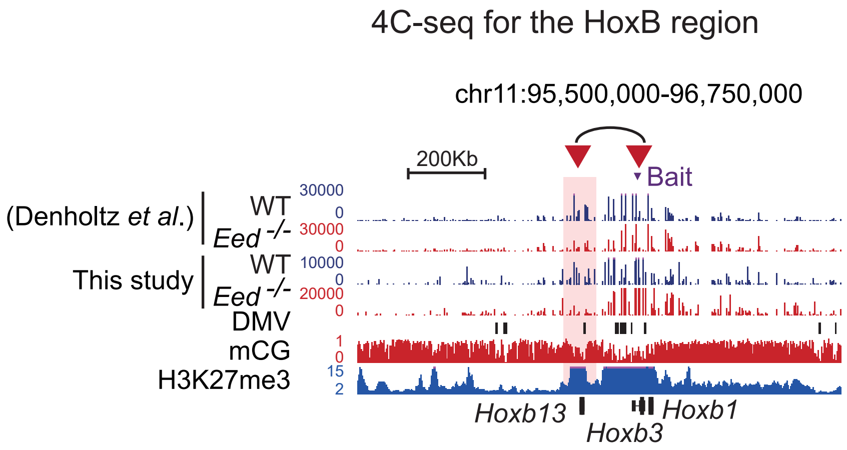
4C-seq for the HoxB region
chr11:95,500,000-96,750,000
200Kb
Bait
(Denholtz et al.)
This study
WT
Eed -/-
DMV
mCG
H3K27me3
Hoxb13
Hoxb1
Hoxb3

Figure S6

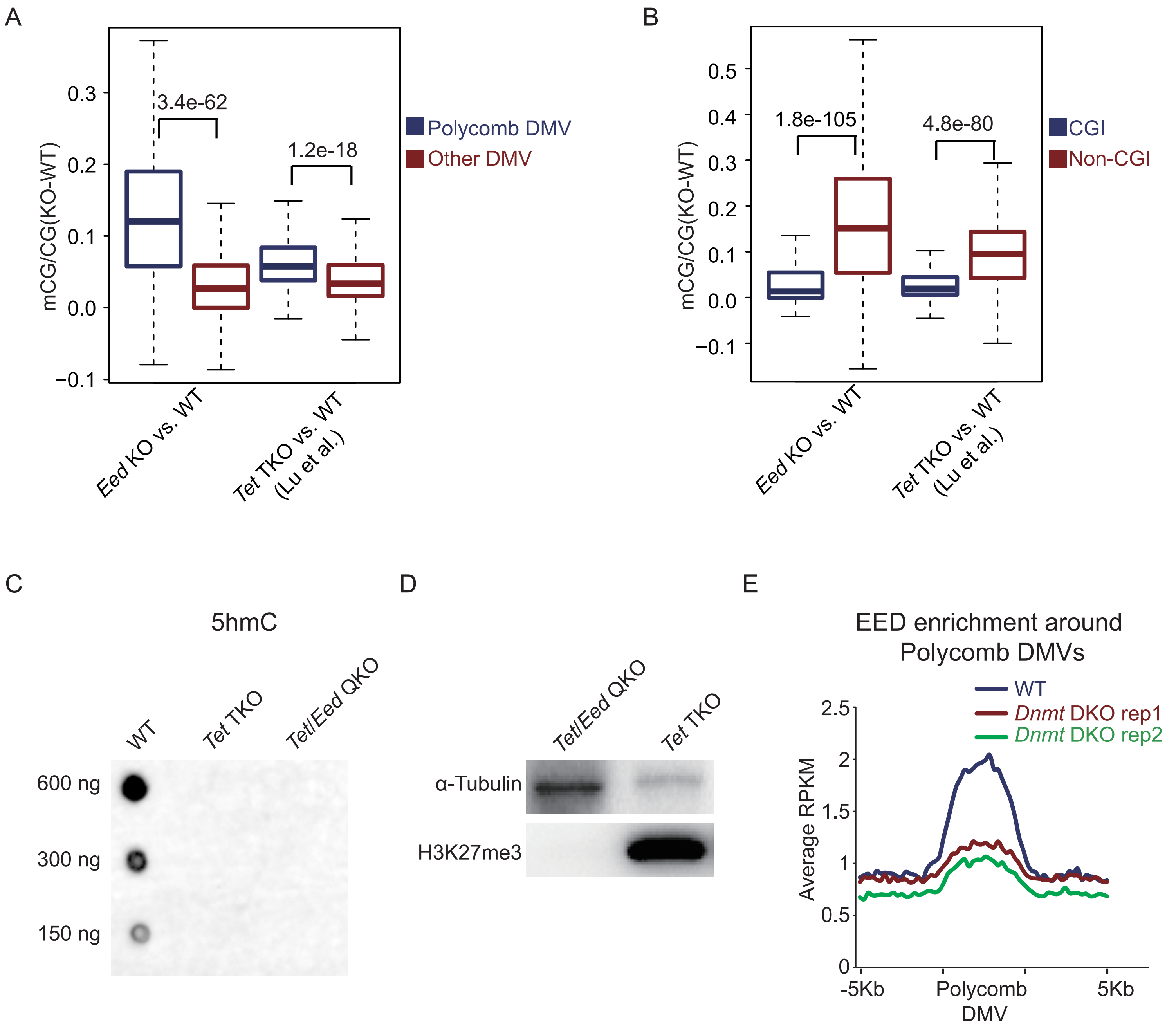
A
mCG/CG(KO-WT)
3.4e-62
1.2e-18
Polycomb DMV
Other DMV
Eed KO vs. WT
Tet TKO vs. WT (Lu et al.)
B
mCG/CG(KO-WT)
1.8e-105
4.8e-80
CGI
Non-CGI
Eed KO vs. WT
Tet TKO vs. WT (Lu et al.)
C
5hmC
WT
Tet TKO
Tet/Eed QKO
600 ng
300 ng
150 ng
D
Tet/Eed QKO
Tet TKO
α-Tubulin
H3K27me3
E
EED enrichment around Polycomb DMVs
WT
Dnmt DKO rep1
Dnmt DKO rep2
Average RPKM
-5Kb
Polycomb DMV
5Kb
